# Feature-specific threat coding in lateral septum guides defensive action

**DOI:** 10.21203/rs.3.rs-6831193/v1

**Published:** 2025-06-12

**Authors:** Dionnet Leandro Bhatti Mazo, Amanda Loren Pasqualini, Sherry Jingjing Wu, Marc Z.C. Berger, Christopher M. Reid, Salvador Ignacio Brito, Shenfeng Qiu, Pat Levitt, Todd Erryl Anthony, Gord Fishell

**Affiliations:** 1 Department of Neurobiology, Harvard Medical School, Boston, MA, USA; 2 Program in Neuroscience, Harvard Medical School, Boston, MA, USA; 3 F.M. Kirby Neurobiology Center, Boston Children’s Hospital and Harvard Medical School, Boston, MA, USA; 4 Departments of Psychiatry and Neurology, Harvard Medical School, Boston, MA, USA; 5 Stanley Center for Psychiatric Research, Broad Institute, Cambridge, MA, USA; 6 Whitehead Institute for Biomedical Research, Cambridge, MA, USA; 7 Department of Basic Medical Sciences, University of Arizona College of Medicine, Phoenix, AZ, USA; 8 Children’s Hospital Los Angeles, The Saban Research Institute, Los Angeles, CA, USA

## Abstract

The ability to rapidly detect and evaluate potential threats is essential for survival and requires the integration of sensory information, with internal state and prior experience. The lateral septum (LS), an inhibitory structure in the limbic forebrain, is thought to integrate these higher-order ‘cognitive’ signals to regulate defensive responses^1,2^. However, the cellular, circuit, and computational mechanisms fundamental to this process remain unknown. Here, we focus on the LS population expressing the type 2 CRH receptor (LS^*Crhr2*^), a neuronal subset shown to be critical for state-dependent behavioral changes and threat responsivity^3–7^. We use a combination of single-cell calcium imaging, molecular sequencing, and circuit dissection to reveal the spatial and functional organization of the cell-types involved, the computations they perform, and the information relayed by their upstream activators. We determined that LS^*Crhr2*^ population activity is required for cue-driven defensive actions by rapidly and dynamically encoding threat representations that predict behavioral outcomes. We find that these threat representations are formed through the convergence of various signals differentially represented among distinct LS^*Crhr2*^ subclasses, which are defined by their molecular features, spatial location, and input architecture. Most importantly, their responses reflect specific afferents from the hippocampus and hypothalamus that preferentially impart cue- and action-related signals, respectively. These findings establish a multifeatured organizational principle that underlies how LS mediates motivated behaviors in response to environmental challenges.

## Introduction

For all animals, the ability to link sensory stimuli with internal states and past experiences is critical for establishing adaptive behavioral responses to environmental cues. This cognitive process enables animals to evaluate the environment and initiate situationally appropriate behavioral and physiological responses. Dysfunction in this ability is detrimental to survival as it hinders prospects for sustenance, mating, and avoiding harm. In humans, such deficits can lead to psychiatric conditions, including anxiety and substance abuse. Over the past century, the LS has emerged as a key regulator of motivated behaviors, including reward seeking, feeding, and social interactions, and most importantly defensive behaviors^2,8–16^. The LS is perhaps best known for the “septal rage” phenomenon, wherein lesions cause hyper-reactivity to innocuous stimuli^17–20^. Paradoxically, these same lesions also impair learned defensive responses to actual threats^17,21,22^. These situationally inappropriate responses suggest a cognitive disconnect between sensory input and action.

Within the LS, CRHR2 signaling is critical for how animals respond to environmental stimuli^23–25^. Moreover, the activity of neurons expressing CRHR2, LS^*Crhr2*^, is crucial for threat responsivity and state-dependent modulation of behavior^6,7^. This neuronal population is well-positioned to integrate diverse types of sensory information due to its input-output circuitry, which primarily involves the hippocampus and hypothalamus^1,7^. The hippocampus chiefly conveys contextual and associative signals, enabling predictive evaluation of environmental stimuli based on past experience^26,27^. In contrast, the hypothalamus conveys motivational signals reflecting internal physiological states, drives, and sensory salience^28^. However, the precise mechanisms by which the LS integrates these modes of information to guide the selection of complex, motivated behaviors remain unclear.

In this study, we employ an instrumental avoidance task together with *in vivo* optogenetics and calcium imaging for manipulating and monitoring the activity of LS^*Crhr2*^ neurons. We find this population is comprised of molecularly diverse cells, whose activity reflects specific afferents and discrete threat-related features. Consequently, this allows the LS^*Crhr2*^ population to collectively contribute to an adaptive stimulus-driven defensive process. This work thus clarifies how LS^*Crhr2*^ cells represent and integrate diverse forms of threat-related information to guide adaptive actions through its computational, molecular, and anatomical properties.

## Results

### LS *Crhr2* population dynamics during active avoidance

Activation of LS^*Crhr2*^ neurons triggers physiological arousal and modulates behavioral threat responses^6,7^, while ablation of these neurons results in behavioral phenotypes reminiscent of the historical observations in lesion studies – most notably, dysfunction in behavioral responsivity to environmental stimuli (**Extended Data Fig. 1**). Furthermore, acute LS^*Crhr2*^ inhibition suppresses learned behavioral and physiological responses to threat (**Extended Data Fig. 2**). To uncover the underlying mechanisms by which LS^*Crhr2*^ contributes to threat processing, we assessed LS^*Crhr2*^ neural dynamics during an instrumental avoidance task called two-way signaled active avoidance (AA) in which mice learn to execute defensive actions to avoid a mild foot-shock in response to an auditory cue. This task is particularly suitable due to its ability to capture distinct threat-related features, including the learning and expression of stimulus-outcome associations, defensive action execution, and trial-to-trial defense decisions.

**Figure 1.**
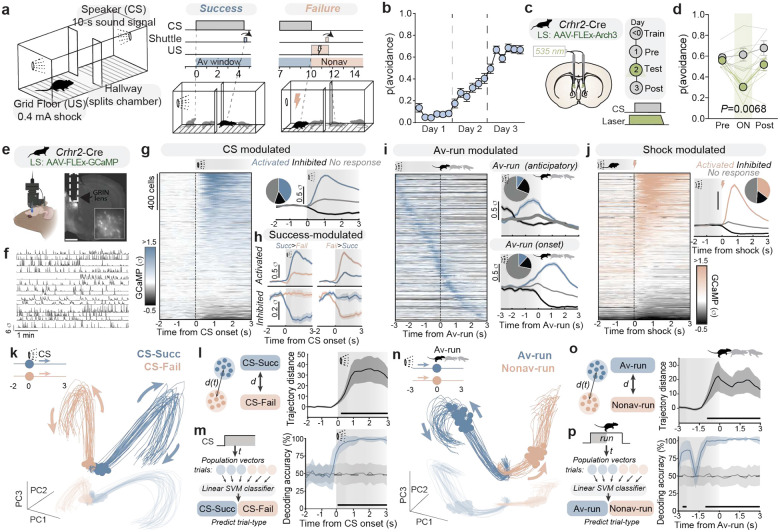
LS *Crhr2* population dynamics represent distinct features of active avoidance. **a**, Schematic of the two-way AA task and typical behavioral responses during success and failure trials. **b**, Probability of successful avoidance in 5-trial blocks across training days (n = 47 mice, One-way ANOVA, *P*<0.0001). Error bars signify mean ± SEM. **c**, Schematic illustrating Cre-dependent viral expression of the inhibitory opsin Archaerhodopsin3.0 and selective photoinactivation of LS^*Crhr2*^ neurons. **d**, Probability of avoidance on the day prior to, during, and after photoinhibition. Photoinactivation of LS^*Crhr2*^ reduced avoidance behavior (n = 6 GFP, 9 Arch3; *P*=0.0096, repeated measure two-way ANOVA; Bonferroni multiple comparisons post-hoc tests, *P*= 0.0068). Error bars signify mean ± SEM. **e**, Experimental strategy for microendoscopic imaging of individual LS^*Crhr2*^ neurons (47 mice, 1654 cells) and example of GCaMP6f expression and implant. Scale bar, 800um. **f**, Representative calcium transients in select LS^*Crhr2*^ neurons. **g–j**, Heatmaps and peri-event time histograms showing distinct subsets of LS^*Crhr2*^ neurons modulated by (**g**) CS onset (sorted by maximum CS response), (**h**) success, (**i**) Av-runs (sorted by time of maximum response), and (**j**) shock onset (sorted by maximum shock response). Insets show averaged activity traces for modulated neurons. Percentages indicate proportion of neurons activated or inhibited defined by AUROC values (see Methods). **k–p**, Neuronal pseudopopulation trajectories (30 per trial type, see Methods) for trial types projected to the first 3 principal components (PC) to (**k–m**) CS onset and (**n–p**) Av-run conditions. Population trajectories diverge based on trial outcome: successful avoidance (CS-Succ, blue) versus failure (CS-Fail, orange), and Av-run versus nonav-run trials. Circles indicate time zero. (**l,o**) Quantification of the normalized distance (*d*) between different trial types over time (mean ± 1 SD, Methods). Thick black lines indicate significantly increased trajectory distance (P < 0.05, permutation test). (**m,p**) Predictive decoding of trial outcomes from LS^*Crhr2*^ activity using linear SVM classifiers (mean ± 1 SD). Thick lines indicate significant decoding accuracies compared to shuffle (P < 0.05, permutation test).

In AA, mice are placed in a two-way shuttle box and presented with auditory cues (i.e. conditioned stimulus: CS) predicting a mild foot-shock (i.e. unconditioned stimulus: US) (Fig. 1a). In response to the CS, mice learn and perform goal-directed avoidance behavior (i.e. avoidance runs: Av-run). Av-run executions occurring within 10 seconds terminate the CS and preclude the shock – these trials are termed success or “CS-Succ” trials. Failure to avoid within 10 seconds results in a shock lasting until the mouse runs to the opposing side – termed failure or “CS-Fail” trials. After just a few days, mice quickly learn the instrumental contingency and perform Av-runs with ~70% success (Fig. 1b). Critically, we find these learned defensive actions require LS^*Crhr2*^ population activity, as photoinhibition during the CS markedly reduces avoidance (Fig. 1c–d, **Extended Data Fig. 2b-j**).

To examine the engagement of the LS^*Crhr2*^ population during AA, we targeted the genetically-encoded calcium indicator, GCaMP6f to these neurons, and a gradient index (GRIN) lens in LS for microendoscopic imaging of single cell activity (Fig. 1e–f). We recorded activity from 1654 individual LS^*Crhr2*^ neurons spanning the entire anatomical axis (**Extended Data Fig. 3a**) across 45 freely-behaving mice during the AA task. After learning the AA task contingencies, many LS^*Crhr2*^ neurons exhibited cue-driven modulation of activity (38.5% increased, 15% suppressed) (Fig. 1g). Within this modulated subset, many neurons displayed biases toward the defense outcome of the trial– either success or failure (Fig. 1h). This suggests that single LS^*Crhr2*^ neurons dynamically respond to threat cues in a way that might influence defense decisions. LS^*Crhr2*^ neurons were also modulated both immediately preceding (i.e. anticipatory; 10% activated) and during (12% activated) Av-runs (Fig. 1i), suggesting a potential role in facilitating the defensive action execution. As expected, a subset of neurons (63% activated, 5.5% inhibited) were also shock-responsive (Fig. 1j). However, the overall neural activity across the LS^*Crhr2*^ population was highly heterogeneous, with individual neuron activity not exclusively associated with a single variable but rather showing varying degrees of correlation with multiple task variables (**Extended Data Fig. 3b-e**). These findings suggest that single-neurons do not have a relationship with discrete task-variables but rather integrate multiple aspects of threat-related information to orchestrate successive steps in cue-driven threat processing and defensive responding.

To identify structure in LS^*Crhr2*^ population activity, we applied dimensionality reduction techniques to generate a low-dimensional embedding of neural data (see Methods) aligned to two key time points – CS onset and avoidance runs^29^. We assessed whether neural activity patterns differed between trial conditions (avoidance success vs. failure) within this neural state space. We hypothesized that if LS^*Crhr2*^ population dynamics are indeed integral for evaluating sensory cues to guide adaptive actions, divergences between the two trial types in response to the cue should emerge. Prior to the CS onset, LS^*Crhr2*^ population activity during both success and failure trial types occupied similar subspaces within the neural state space across both trial types. However, immediately following CS onset, LS^*Crhr2*^ neural trajectories rapidly diverged depending on whether a defensive action was initiated seconds later in the CS period (i.e., avoidance success or failure) (Fig. 1k–l). Strikingly, LS^*Crhr2*^ population activity within just one second of cue presentation was highly predictive of future avoidance success before any action was initiated (Fig. 1m, **Extended Data Fig. 3g**). Thus, threat cue representations in LS^*Crhr2*^ population activity them-selves contain information about the decision to initiate action that is realized seconds later. This finding suggests that information relevant for evaluating potential threats (e.g., stimulus-salience) may be integrated by the LS^*Crhr2*^ population immediately upon the cue onset to guide action selection. Interestingly, a second divergence in neural trajectories occurs immediately prior (~1.5 seconds) to the avoidance run initiation (Fig. 1n). These neural trajectories began to diverge until the onset of the avoidance run, at which point the trajectories began converging back toward their initial subspace (Fig. 1o). LS^*Crhr2*^ population activity was also highly predictive of the defensive outcome immediately (as early as ~1.5 seconds) preceding defensive action initiation (Fig. 1p, **Extended Data Fig. 3h**) but not running itself (**Extended Data Fig. 3i**). These data indicate that LS^*Crhr2*^ neurons dynamically encode future defensive responses at two temporally distinct stages of the threat representation. First, at the cue onset, which we interpret to reflect a role for LS^*Crhr2*^ in sensory evaluation aimed to support decision-making^30^. And second, immediately prior to action initiation, which may reflect motivational salience that guides avoidance.

### Functional classes of LS *Crhr2* neurons are spatially organized and represent distinct threat-related features

Threat processing requires the appropriate detection and evaluation of sensory stimuli to dictate suitable defensive decisions^31,32^. This process depends upon both top-down influences (representing prediction and experience) and bottom-up influences (representing motivational salience). We hypothesized that task-relevant structure in LS^*Crhr2*^ population dynamics might emerge from single-neuron representations of these distinct threat-related features. To directly assess their contributions to threat cue and action representations we examined the activity of individual cells by constructing a low-dimensional activity space that maximally separates response vectors in CS-Success and CS-Failure trials termed the coding direction (CD, see Methods)^33^. Each CD and principal component (PC) represent separable and orthogonal dimensions of the neural population dynamics (**Extended Data Fig. 4–5**). We found that the cue-, cue-outcome, prior-to-action, and action dimensions are each topographically organized within LS anatomical space (**Extended Data Fig. 4e and k**, Fig.5e and j). Consistent with this, perturbations of discrete anatomical subregions in LS can have varying impacts on behavior, indicating possible cell-types with distinct threat-related functions^20,34,35^.

**Figure 2.**
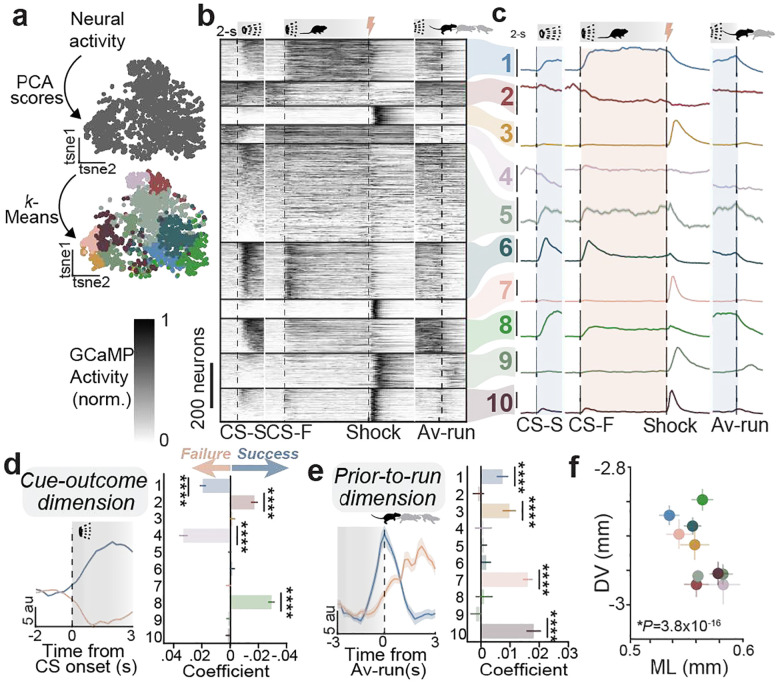
Task-related activity dynamics define functional clusters of spatially organized LS^*Crhr2*^ neurons. **a**, Schematic showing clustering approach based on PCA scores from single-neuron GcaMP activity during AA. **b**, Heatmap of normalized calcium activity from all LS^*Crhr2*^ neurons (47 mice, 1654 neurons) grouped by identified functional clusters (k-means clustering, clusters labeled 1–10, n = [1] 181, [2] 106, [3] 82, [4] 81, [5] 429, [6] 246, [7] 83, [8] 150, [9] 151, [10] 145 neurons). **c**, Average activity profiles for each cluster aligned to distinct task epochs: CS success (CS-S), CS failure (CS-F), shock, and Av-run initiation. Each color signifies distinct cluster identity. **d**, Left, population activity projected onto the Cue-outcome dimension aligned to CS onset (coding direction, CD, see Methods) across trial types. Right, CD coefficients across clusters. Select clusters preferentially represent the Cue-outcome dimension, highlighting significant predictive activity at CS onset (one sample t-test and Wilcoxon test). **e**, Left, population activity projected onto the Prior-to-run dimension aligned to run onset (PC3, see Methods) across trial types. Right, PC3 coefficients across clusters. Select clusters preferentially represent the Prior-to-run dimension (one sample t-test and Wilcoxon test), highlighting significant predictive activity preceding action initiation. Trial types: successful avoidance (blue) versus failure (orange). **f**, Spatial topography of functional LS^*Crhr2*^ clusters represented by dorsoventral (DV) and mediolateral (ML) coordinates (mean ± SEM; *P* = 3.8 × 10^−16^, ANOVA). *P < 0.05, ** *P* < 0.01, ***P < 0.001, ****P < 0.0001.

To determine how these representations are stratified among distinct functional classes of LS^*Crhr2*^ neurons, we employed a clustering approach (Fig. 2a, **Extended Data Fig. 6a-d**, see Methods) to categorize LS^*Crhr2*^ neurons based on their activity patterns during the AA task. This method revealed a remarkable diversity in activity-defined LS^*Crhr2*^ subclasses. Neuronal dynamics among these clusters were distinguished by the following characteristics: 1) whether the neurons were activated or inhibited by the CS, 2) differences in CS response magnitude between success and failure trials, 3) transience versus persistence of CS-evoked responses, 4) shock responsivity, 5) responses to “safe zone” entry or escape completion, and 6) Av-run responses (Fig. 2b–c, **Extended Data Fig. 6e-n**). Interestingly, these activity-defined LS^*Crhr2*^ subclasses are differentially represented by functional dimensions associated with stimulus-outcome pairings, defensive outcomes, avoidance action, or stimulus-salience (**Extended Data Fig. 6o-v and 7**). Specifically, clusters 1,2,4, and 8 contribute to the ‘cue-outcome’ variable, while clusters 1,3,7, and 10 represent an up coming ‘action’ variable (Fig. 2d–e). Furthermore, individual clusters are topographically organized (Fig. 2f, **Extended Data Fig. 8**). These findings suggest that the spatial organization and functional specialization of LS^*Crhr2*^ subclasses control the processing of threat-related cues via the representation of ‘top-down’ cue-outcome variables and ‘bottom-up’ action-related signals. We posit that these representations are the key substrate that enables the LS to orchestrate precise defensive behaviors in response to environmental challenge.

### Molecular and anatomical features define LS *Crhr2* function

**Figure 3.**
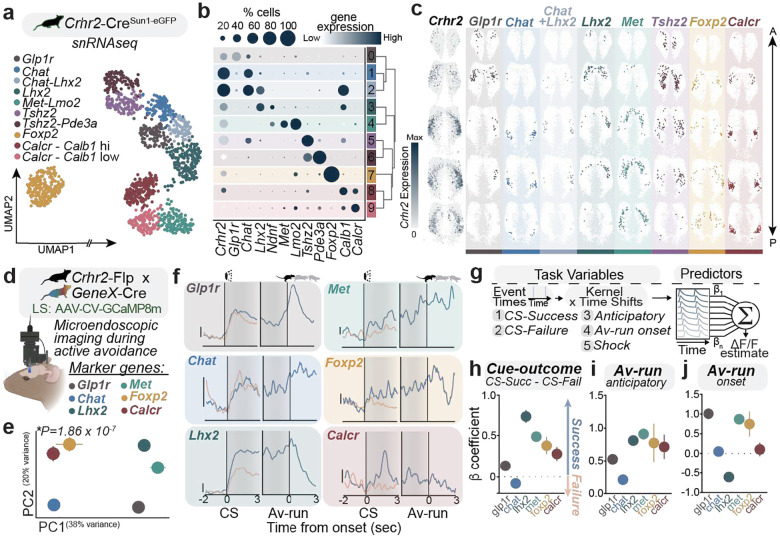
Transcriptomic identity defines spatially and functionally organized LS^*Crhr2*^ subclasses. **a**, UMAP visualization of snRNAseq data showing 10 distinct molecular subclasses of LS^*Crhr2*^ neurons identified by unique gene expression. **b**, Dot plot illustrating the percentage of cells expressing key marker genes and their relative expression levels across identified subclasses. **c**, Spatial mapping of molecular subclasses within the LS, illustrating distinct anatomical distributions of each LS^*Crhr2*^ subtype (*Glp1r*, *Chat*, *Lhx2*, *Met*, *Tshz2, Foxp2*, *Calcr*). **d**, Schematic illustrating intersectional genetic strategy combining *Crhr2*-Flp with Cre-recombinase mouse lines for each marker gene (GeneX-Cre) for subtype-specific expression of GCaMP8m and microendoscopic calcium imaging. Each color denotes subtype. **e**, Mean ± SEM values for each molecular subtype across the first 2 PCs in a low-dimensional space representing neural activity patterns across epochs of AA task variables (CS-Success, CS-Failure, Shock, and Av-run). *Glp1r:* n=6 mice, 252 neurons; *Chat:* n=10 mice, 194 neurons; *Lhx2*: n=10 mice, 262 neurons; *Met*: n=2 mice, 70 neurons; *Foxp2:* n=2 mice, 23 neurons; *Calcr:* n=2 mice, 26 neurons. **f**, Representative calcium activity traces aligned to CS-Succ (blue), CS-Fail (orange), and Av-run from molecularly defined subclasses. Scale bars begin at 0 and represent 0.5 SD from the mean. LS^*Crhr2*^ subtypes each display differential responses to each variable. **g**, Schematic for generalized linear model (GLM) predicting neural activity from AA task variables. **h–j**, GLM-derived β coefficients indicating differential representation of (**h**) cue-outcome signals (calculated by subtracted CS-Fail from CS-Succ β coefficients), (**i**) anticipatory Av-run, and (**j**) Av-run onset across molecular subclasses (mean ± SEM). Each LS^*Crhr2*^ subtypes has a unique relationship across the AA task variables.

Consistent with specific engagement of LS^*Crhr2*^ neurons, chemoarchitectural differences within LS have been identified and proposed to dictate distinct function^2,3^. In line with this idea, several groups have identified putative subsets of LS neurons that may underlie distinct behavioral phenomena^13,36,37^. However, a detailed survey linking functional types with molecular expression has not been performed. We posited that within the LS^*Crhr2*^ population, molecular identity delineates the functional organization. We thus explored the transcriptional diversity among the LS^*Crhr2*^ population using single-nuclei RNA sequencing (snRNAseq). Contrary to recent reports that treat LS^*Crhr2*^ as one unified population of neurons^38,39^, our data resolved 10 transcriptionally unique subclasses of LS^*Crhr2*^ neurons defined primarily by their co-expression with marker genes *Glp1r, Chat, Lhx2, Met, Calcr, Foxp2, or Tshz2* (Fig. 3a–b, **Extended Data Fig. 9**), each of which exhibited a unique spatial distribution (Fig. 3c, **Extended Data Fig. 10a**) and relationship to previously defined LS subtypes (**Extended Data Fig. 10b-e**)^40^. It is possible that the representations of distinct threat-related features may be attributed to molecularly-defined subpopulations of LS with discrete topography. We thus hypothesized that these LS^*Crhr2*^ subtypes may display distinct activity patterns with unique function that are consistent with the LS^*Crhr2*^ subsets described previously (Fig. 2).

Consequently, we sought to establish a relationship between threat-related functions in active avoidance and molecular subclasses. We thus used genetic approaches to target the various transcriptomic classes of LS^*Crhr2*^ neurons. We targeted the calcium indicator GCaMP8m to several key subtypes including *Glp1r, Chat, Lhx2, Met, and Calcr* using an intersectional Cre/Flp strategy^41^ (i.e. by combining *Crhr2*^*flp*^ and *GeneX*^*cre*^) (Fig. 3d, **Extended Data Fig. 11a-g**). We then subjected these mice to AA while recording individual neurons within each subtype across mice. We first clustered the neural activity across all subtypes and performed dimensionality reduction along the time axis. The low-dimensional space indicated significant differences among the subtypes, while also demonstrating similarities among groups such as *Chat* and *Glp1r* as well as *Foxp2* and *Met* (Fig. 3e). We found that if we cluster individual neurons independent of their molecular identity (as performed in Fig. 2), clusters are largely constrained to single molecularly-defined subtypes (**Extended Data Fig. 11h-l**) suggesting that molecular identity largely fits to function. By aligning the neural activity to two key time points – CS and Av-run onsets, we assessed whether neural activity patterns differed between trial-types (avoidance success or failure). We found that all subtypes were significantly modulated by both the CS and Av-run, as well as shock, but with different dynamics depending on trial type (Fig. 3f, **Extended Data Fig. 11m-n**). To directly quantify and distinguish the contribution of each stimulus and behavioral variable in active avoidance, we built a generalized linear model (GLM) to fit and predict the activity of each neuron across subtypes (Fig. 3g, **Extended Data Fig. 11o**). We discovered that the weights of select threat-related variables were differentially represented across the subtypes (Fig. 3g, **Extended Data Fig. 11p-t**). Specifically, ‘cue-outcome’-related signals – defined as the difference between CS-S and CS-F b coefficients (which reflect the strength and direction of each variable’s influence on neural activity) – were represented across all subtypes, albeit with distinct strengths and directions (Fig. 3h). For example, the ‘cue-outcome’ variable was best represented in the *Lhx2* subtype neurons, where a clear increase in CS-onset responsivity occurred in success trials relative to failure, while in *Chat* subtype neurons the opposite trend was observed. Although the representation of anticipatory Av-run signals (i.e. 1 second *prior-to-action*) was also represented across subtypes, the magnitude of modulation was greatest in the *Met*, *Lhx2*, *Foxp2*, and *Calcr* subtypes (Fig. 3i). Av-run *onset* representations (1 second following onset), however, were greatest in *Glp1r* subtype neurons, followed by *Foxp2* and *Met* (Fig. 3j). Interestingly, each molecularly-defined subtype differentially represented functional dimensions associated with ‘cue-outcome’, upcoming action, and action execution (**Extended Data Fig. 12 and 13**). Together, these data suggest that molecular identity defines LS^*Crhr2*^ subsets with topography and functional associations. Specifically, select subtypes differentially encode aspects of threat-related variables, which may arise through differential convergence of afferent signals encoding of cue- and action-related features.

### Molecularly distinct LS *Crhr2* populations receive specific threat-related information from select afferents

**Figure 4.**
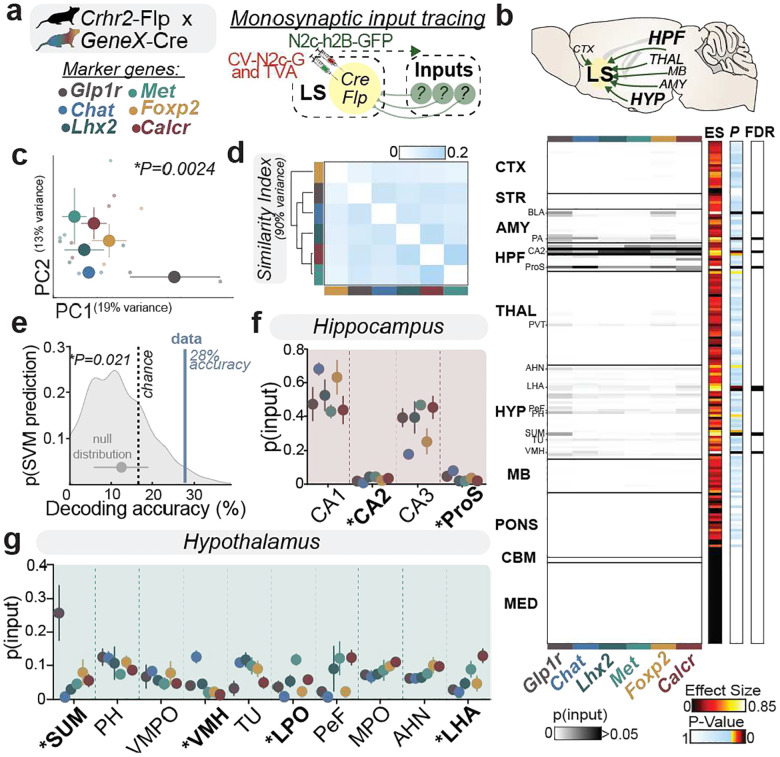
Distinct afferent organization among LS^*Crhr2*^ subclasses. **a**, Schematic illustrating intersectional strategy using *Crhr2*-Flp and GeneX-Cre mice for monosynaptic rabies tracing to identify inputs to each molecular subclass. **b**, Anatomical summary of afferent inputs to LS^*Crhr2*^ subclasses from cortical (CTX), striatal (STR), amygdala (AMY), hippocampal formation (HPF), thalamic (THAL), hypothalamic (HYP), midbrain (MB), pontine (PONS), cerebellar (CBM), and medullary (MED) regions. Heatmap shows proportion of all inputs; Right, statistical significance (single comparison *P*-values and false discovery rates (FDR); effect size indicated; n=2–3 mice per subtype; see Methods). **c**, Mean ± SEM values for each molecular subtype across the first 2 PCs in a low-dimensional space distinguishing afferent input patterns across molecular subclasses (one-way ANOVA, *P* = 0.0024). **d**, Heatmap of similarity index of afferent connectivity between subclasses highlighting closer relationships among certain subtypes in the attached dendrogram. **e**, Decoding accuracy distribution showing classifier performance distinguishing subtypes based on input connectivity (vertical dashed line, chance level; *P* = 0.021, permutation test). Gray: null accuracy distribution. Blue: real data accuracy. **f**, Proportion of inputs from the four most innervating HPF subregions showing subtype-specific targeting from CA1, CA2, CA3, and prosubiculum (ProS) (one-way ANOVA, *P<0.05). **g**, Proportion of inputs from the ten most innervating hypothalamic subregions illustrating preferential innervation from supramamillary nucleus (SuM), ventromedial hypothalamus (VMH), lateral preoptic area (LPO), and lateral hypothalamus (LHA) to specific subclasses, as well as targeting from the posterior hypothalamus (PH), ventromedial preoptic area (VMPO), tuberal nucleus (TU), perifornical nucleus (PeF), medial preoptic (MPO), and anterior hypothalamic area (AHN) (one-way ANOVA, *P<0.05).

The distinct engagement of specific LS^*Crhr2*^ populations likely reflects their afferent inputs^2,42^. For example, it is well established that distinct neuromodulators target discrete anatomical domains within the LS^3^. LS^*Crhr2*^ neurons are most prominently innervated by a combination of hypothalamic and ventral hippocampal regions (with the exception of dorsal CA2) (**Extended Data Fig. 14**)^2,6,7^. While a prevalent theory suggests that LS may serve as a relay for top-down ‘cognitive’ inputs to hypothalamic structures^2,16,43–46^, the hypothalamus also provides robust reciprocal innervation to the LS^1^. An intriguing idea is that ascending hypothalamic inputs serve as bottom-up signals, whereas hippocampal inputs serve as top-down processing units. In this context, the hypothalamus relays motivational- or stimulus-salience information that may influence action execution, while the hippocampus provides ‘cognitive’ predictive information relating stimulus-outcome associations that inform decisions. In fact, bottom-up ‘salience’ and top-down ‘cognitive’ signals have been proposed to underlie adaptive attentional control of both threat and visual processing in humans^47–50^. We posited that spatially and molecularly organized functional classes of LS^*Crhr2*^ neurons may receive biased hippocampal and hypothalamic input which in turn shape functional specializations in single neurons. To examine the specific inputs received by these LS^*Crhr2*^ subpopulations, we used an intersectional strategy (described previously in Fig. 3) to target specific subclasses. This was achieved using a combined Flp/Cre-activated helper viruses and CVS-N2cDG modified rabies virus expressing h2B-GFP to label monosynaptic inputs to each LS^*Crhr2*^ subclass followed by serial 2P tomography and automated brain-wide cell counting (Fig. 4a). We found that the hippocampus and hypothalamus robustly innervate all LS *Crhr2* populations, regardless of the subclass targeted (Fig. 4b, **Extended Data Fig. 14h**-**m**). In addition, certain subtypes displayed specific connectivity with select brain structures within the amygdala. For instance, the basolateral amygdala (BLA) selectively targets *Glp1r*- and *Foxp2*-expressing subtypes, while the posterior amygdala (PA) selectively targets the *Chat*-expressing subtype (**Extended Data Fig. 14d)**. In contrast, most *LS*^*crhr2*^ subtypes received afferents from the paraventricular area of the thalamus (PVT), a higher-order thalamic structure (**Extended Data Fig. 14e and n**). To determine select differences between subtypes, we reduced the dimensionality of their afferent connectivity using PCA (Fig. 4c). Using the PCs that explain 90% of the variance, we found that the subtypes indeed display differential patterns of afferent connectivity. Though notably, select subtypes displayed more similarity with each other than others (Fig. 4d). For example, *Lhx2-, Calcr-, and Met*- expressing subtypes, as well as *Chat*- and *Glp1r*- expressing subtypes each respectively displayed between-group similarities reminiscent of their transcriptomic relationships (Fig. 3). To further determine the strength of the differences between the inputs across subtypes, we trained a decoder to distinguish subtypes based on the proportion of their inputs across the brain and found that the decoder performed better than chance (Fig. 4e).

Given the hippocampus and hypothalamus are the most abundant LS^*Crhr2*^ afferents, we hypothesized that the information relayed from these two structures represent the most critical signals relayed to the LS for active avoidance. We thus sought to more precisely quantify the proportion of inputs from hippocampal and hypothalamic structures to each subtype. Within the hippocampal formation (HPF), the ventral CA1, CA2, CA3, and prosubiculum (ProS) most abundantly targeted LS^*Crhr2*^ subtypes – the CA2 displayed more innervation of the *Met- and Glp1r*- expressing subtypes while the ProS preferentially targeted the *Chat*-expressing subtype (Fig. 4f). Within the hypothalamus (HYP), the supramamillary nucleus (SuM), posterior hypothalamus (PH), multiple preoptic nuclei, ventromedial hypothalamus (VMH), anterior hypothalamus (AHN), and the lateral hypothalamus (LHA) collectively provided the largest number of afferents targeting the LS^*Crhr2*^ subtypes—with the SuM, VMH, lateral preoptic (LPO), and LHA preferentially targeting the *Glp1r*-, *Chat*-, *Met*-, and *Calcr*-expressing subtypes, respectively (Fig. 4g). Importantly, upon further examining the precise substructures in HPF and HYP that provided the bulk of the afferents to the LS^*Crhr2*^ cells, we found that these arise from ventral, not dorsal hippocampal structures (with the exception of CA2) and the lateral hypothalamic neurons located specifically within the subfornical area of the LHA (LHAsf) (**Extended Data Fig. 14**).

We next aimed to validate the contribution of hippocampal inputs in imparting ‘top-down’ cue-outcome information and hypothalamic inputs in relaying ‘bottom-up’ action-related signals. For example, as the *Glp1r* subtype receives the most input from the SuM and is highly modulated by Av-run onset (Fig. 3f and 4j), we predicted that the SuM relays an action-related signal. Similarly, as the LHA sends the most input to Met, Calcr, and Foxp2, which are each modulated by anticipatory Av-run (Fig. 3f and 4i), we predicted that the LHA may relay upcoming avoidance signals.

**Figure 5.**
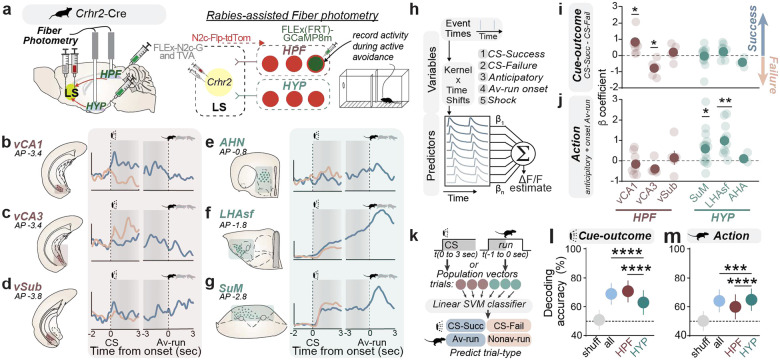
Hippocampal and hypothalamic afferents relay distinct threat-related signals to the LS^*Crhr2*^ population. **a**, Schematic illustrating rabies-assisted fiber photometry strategy to selectively express GCaMP8m in hippocampal (HPF) and hypothalamic (HYP) inputs projecting to LS^*Crhr2*^ neurons and record their bulk calcium activity. **b–d**, Mean calcium activity traces aligned to CS-Succ (blue), CS-Fail (orange), and Av-run fiber from hippocampal inputs: ventral CA1 (vCA1, **b**, n= 7 mice), ventral CA3 (vCA3, **c**, n= 5 mice), and ventral subiculum (vSub, **d**, n= 5 mice). **e–g**, Corresponding recordings from hypothalamic inputs: anterior hypothalamic nucleus (AHN, **e**, n= 3 mice), lateral hypothalamic subfornical area (LHAsf, **f**, n= 10 mice), and supramamillary nucleus (SuM, **g**, n= 15 mice). Scale bars begin at 0 and represent 0.5 SD from the mean. **h**, GLM schematic for predicting afferent neural activity from from AA task variables. **i–j**, GLM-derived β coefficients demonstrating differential representation of (**i**) cue-outcome signals predominantly in hippocampal inputs, and (**j**) action-related signals predominantly in hypothalamic inputs (One-sample t-test and Wilcoxon test). Dark circles represent mean ± SEM; lighter circles represent individual mice. **k**, Schematic depicting linear SVM classifiers predicting trial outcomes based on afferent neural activity at CS onset (cue-outcome) or preceding Av-run (action). **l–m**, Decoding accuracy showing that (**l**) hippocampal inputs more accurately predict trial outcomes at CS onset, whereas (**m**) hypothalamic inputs better predict outcomes related to Av-run onset (mean ± SEM). Red: HPF subregions. Green: HYP subregions. *P < 0.05, **P < 0.01, ***P < 0.001, ****P < 0.0001).

To determine whether hippocampal and hypothalamic inputs signal these distinct threat related features, we targeted a modified CVS-N2cΔG rabies virus expressing Flp-tdTomato to LS^*Crhr2*^ neurons to allow for Flp-dependent molecular expression within LS^*Crhr2*^ afferents^51,52^ (Fig. 5a). We expressed Flp-dependent GCaMP8m within three hypothalamic and three hippocampal subregions including the AHN, LHAsf, SuM, ventral CA1 (vCA1), ventral CA3 (vCA3), and ventral subiculum (vSub) (**Extended Data Fig. 15a)**. Using *in vivo* fiber photometry to examine the activity of each input during AA, we found that response dynamics to the CS, shock, and Av-run were distinct across all these inputs (Fig. 5b–g, **Extended Data Fig. 15b-i**). Specifically, vCA1 and vCA3 were each modulated by the CS, preferentially responding to the CS during success and failure trials, respectively. Notably, none of the three hippocampal structures showed modulation by the Av-run (Fig. 5b–d). In the hypothalamus, both LHAsf and SuM were activated by the CS, but were differentially modulated by the Av-run – LHAsf activity increasingly ramped its activity throughout the CS-S until the Av-run was executed, while SuM activity increased specifically at Av-run onset (Fig. 5f–g). To directly quantify and distinguish the contribution of each stimulus and behavioral variable in active avoidance, we built a GLM to fit and predict the activity of each afferent (Fig. 5h, **Extended Data Fig. 15j-p**). We discovered that the weights of specific threat-related variables were preferentially represented in either the hippocampus or hypothalamus. Specifically, ‘cue-outcome’-related signals – defined as the difference between CS-S and CS-F b coefficients (as defined in Fig. 3g–h) – were best represented in hippocampal regions such as the vCA1 and vCA3 (Fig. 5i, **Extended Data Fig. 15k-l**), whereas action-related signals – defined as the sum of anticipatory and onset Av-run b coefficients – were more prominent in hypothalamic structures such as the LHAsf and SuM (Fig. 5j, **Extended Data Fig. 15m-n**). We then trained two decoders to predict trial outcome (avoidance success or failure): one based on afferent activity at CS onset (Cue-outcome) and another based on activity immediately preceding the Av-run (Action) (Fig. 5k). In both cases, using data from all regions, hippocampus, or hypothalamus individually yielded prediction accuracies above chance. However, the responses for CS-onset and Av-run nicely parcellated to each of these structures. When the CS-onset decoder relied solely on hippocampal or all data, it resulted in better accuracy than with hypothalamic data alone (Fig. 5l), whereas when the Av-run decoder was restricted to hypothalamic or all data, it resulted in better predictions than hippocampal data alone (Fig. 5m). These data suggest that functional connectivity differences between LS^*Crhr2*^ subtypes and hippocampal and hypothalamic regions may subserve the differential encoding of threat-related signals within the LS. Thus, we conclude that the LS^*Crhr2*^ population forms diverse threat representations through the convergence of top-down ‘cognitive’ and bottom-up ‘action’ signals.

## Discussion

In this study, we identify the molecular, anatomical, and functional organization of the LS involved in threat processing. We discovered computational and organizational principles that underlie how this structure is linked to motivated behaviors. These findings demonstrate its capacity to evaluate environmental stimuli to guide adaptive defenses. Specifically, we show that this is mediated by LS^*Crhr2*^ subtypes that integrate cue- and action-related sensory information relayed from the hippocampus and hypothalamus.

Remarkably, the LS seems unique in this capacity. Other brain regions involved in defensive behavior such as the BLA^53^, PVT^54^, striatum^55^, and frontal cortex^29^ do not perform these computations during conditioned stimulus presentations. For example, while the frontal cortex can also predict upcoming avoidance, it does so independently of the cue-evoked population dynamics and does not differentiate defense outcomes^29^. This suggests that while frontal cortex may link cues to action, the LS acts upstream to couple environmental cues to experience and internal state. Indeed, given that the LS^*Crhr2*^ neurons are polysynaptically connected to frontal cortex^7^, it places the LS in the unique position of guiding cortical action representations.

LS^*Crhr2*^ neurons appear central to stress-related behavioral modulation^6^. While our results outline the computations performed within this neuronal population, an important next step will be to explore the precise mechanisms by which the CRH-related neuromodulatory system influences this process. It is conceivable, for instance, that stress or threat exposure shifts the balance of functional input between the hypothalamus and hippocampus by altering LS neuronal excitability via CRHR2-mediated signaling. Indeed, maladaptive signaling between bottom-up sensory processing and top-down cognitive control has been proposed as a model for anxiety-related disorders^48,56^, where bottom-up signaling becomes disproportionately strong relative to top-down regulation. In this disease context, bottom-up hypothalamic salience signals would be enhanced and drive defensive actions in response to otherwise non-threatening stimuli.

Our findings also open intriguing avenues regarding intraseptal microcircuitry. Although the LS is traditionally viewed as a tonic inhibitor of downstream behavioral outputs, our results suggest that discrete neuronal subpopulations might shape behavior through population coding mechanisms. Such coding could potentially arise from reciprocal inhibition or modulation among subtypes. Clarifying these detailed microcircuit relationships will be essential in fully understanding the LS’s role in behavioral selection and modulation under both healthy and pathological conditions.

The organizational principles identified in this study are likely generalizable beyond threat contexts and may apply broadly to motivated behaviors. For instance, within reward contexts, LS neurons likely evaluate sensory cues predicting positive outcomes to facilitate reward-seeking behavior^14,39^. Similarly, in social contexts, LS processing of chemosensory and contextual cues likely guide social approach, avoidance, or territory-related actions^11–13,57,58^. Dysfunction within LS, as demonstrated by “septal rage”, would therefore disrupt the brain’s ability to adaptively link experience and internal state with sensory stimuli, resulting in inappropriate behavioral responses. Our study thus provides a broader framework for understanding these complex behaviors by revealing how isolated features of sensory and behavioral processing are represented and computed within the LS.

Taken together, our study provides a novel perspective on the role of the LS, highlighting how its multifeatured organization allows higher order processing of sensory cues to facilitate defensive behavior. This conceptual framework not only deepens our understanding of threat processing but also sets a foundation for future studies exploring LS involvement in various forms of adaptive and maladaptive behavioral modulation.

## Methods

### Animals

All animal care and experimental procedures were approved by the Institutional Animal Care and Use Committee at Harvard Medical School and Boston Children’s Hospital. Male FVBB6 F1 hybrid Crhr2 α -eGFPCre^6^, Crhr2-IRES-Cre^59^ (JAX stock #033728), and Crhr2α Flp (generated for this paper) crossed with Lhx2-CreER^60^ (JAX stock #036293), ChAT-IRES-Cre(Δneo)^61^ (JAX #031661), Glp1r-IRES-Cre^62^ (JAX stock #029283), Met-iCre (this paper), Calcr-IRES-Cre (generated for this paper), Calcr-Cre^63^ (JAX stock #037028), or Foxp2-IRES-Cre^64^ (JAX stock #030541) (3–6 months of age) were used. Animals were maintained on a 12 hour reverse light/dark cycle at least one week prior to and throughout experiments, with free access to food and water ad libitum.

### *Generation of Crhr2α*-Flp BAC transgenic mice

*Crhr2*-Flp mouse was generated using an identical procedure as previously reported for the generation of the *Crhr2*-GFPCre BAC transgenic mouse^6^. A mouse bacterial artificial chromosome (BAC clone RP23–78P13) containing the entire *Crhr2* gene was treated and prepared for the generation of transgenic mice. Mice were maintained with a FVB strain background.

### *Generation of Met-iCre* knock-in mice

The *Met*-iCreERT2 knock-in mouse line was generated by Biocytogen using CRISPR/Cas9-mediated homologous recombination. A targeting vector was constructed to insert a P2A-iCreERT2 cassette in-frame into exon 21 of the target gene (NM_008591). sgRNAs were designed and screened for efficiency. Cas9 mRNA, sgRNA, and the targeting vector were microinjected into C57BL/6N zygotes. Founder mice were identified by junction PCR and confirmed by Southern blot to verify correct targeting and exclude random integration events. Positive F0 mice were bred with wild-type C57BL/6N mice to establish the F1 generation, and heterozygous offspring were confirmed by genotyping. This knock-in enables tamoxifen-inducible Cre recombinase expression under the control of the endogenous promoter without affecting endogenous Met expression.

### *Generation of Calcr-IRES-Cre* knock-in mice

The *Calcr*-IRES-Cre knock-in mouse was generated by inserting an internal ribosome entry site (IRES) followed by a Cre recombinase coding sequence into the 3’ untranslated region (UTR) of the Calcr gene locus. This design allows bicistronic expression of both the endogenous calcitonin receptor (Calcr) and Cre recombinase under control of the native Calcr promoter, ensuring spatial and temporal fidelity of Cre expression.

To confirm successful targeting and germline transmission of the knock-in allele, genotype-confirming PCR was performed using locus-specific primers flanking the insertion site. PCR amplicons were sequenced by next-generation sequencing (NGS) at the MGH DNA Core. Sequence alignment of PCR products against the expected knock-in construct and wild-type genomic sequences verified precise insertion of the IRES-Cre cassette without unintended mutations or rearrangements. Positive F0 mice were bred with wild-type C57BL/6N mice to establish the F1 generation, heterozygous offspring were confirmed by genotyping and bred to homozygosity.

### Stereotaxic surgeries

Mice were anesthetized with isoflurane (4% for induction, 1–2% thereafter) and placed on a stereotaxic frame (Kopf, Germany). The skull was exposed and holes were produced using a hand-held drill. Pressure injections were made by backfilling virus into glass capillaries to be delivered at a rate of 60 nl per minute using a nanoliter injector (Nanoliter 2000, World Precision Instruments) controlled by an ultra microsyringe pump (Micro4, World Precision Instruments); the injector was withdrawn at least 3 min after viral injection completion. Injections were targeted using coordinates based on Paxinos and Franklin mouse brain atlas: relative to bregma in mm; LS (AP +0.6, ML ±0.55, DV-2.92 to −3.22); SuM (AP-2.85 ±0.20, DV-4.7 to −4.95); LHA (AP-1.7 ML±1.1, DV-5.05 to −5.35); vCA1 (AP-3.30 ML+3.4 DV-3.1 to −3.4); vCA3 (AP-3.30 ML+3.4 DV-3.1 to −3.4), vSub (AP-3.80 ML+3.4 DV-3.5 to −4.0). Fiber optics for photometry (400 μm, unilateral) or optogenetics (200 μm, bilateral) and GRIN lens for microendoscopic calcium imaging (500 μm, unilateral) were implanted to meet the following coordinates in Paxinos: relative to bregma in mm; LS (AP +0.3 to 0.6, ML ±0.55, DV-2.7 to −3.2); SuM (AP-2.85 ±0.20, DV-4.8); LHAsf (AP-1.7 ML±1.1, DV-5.15); vCA1 (AP-3.30 ML+3.4 DV-4.2); vCA3 (AP-3.30 ML+2.9 DV-3.5); vSub (AP-3.80 ML+3.4 DV-4.0).

#### Microendoscopic Imaging

For microendoscopic imaging, *Crhr2*-Cre mice were injected with 150nl of AAV5-CAG(del)-GCaMP6f-FLEx(loxP) and/or AAV9-CAG(del)-GCaMP6f-FLEx(loxP) (Addgene, 100835). *Crhr2* subtypes were intersectionally targeted using *Crhr2*-Flp and Cre allele mouse crosses with 150nl of AAV5-hSyn-CV-GCaMP8m and/or AAV9-hSyn-CV-GCaMP8m (plasmid provided by Lindsay Schwarz; generated by BCH viral core). Cortical tissue directly above the LS was aspirated and cleaned using sterile saline solution. 3 anchor screws were secured to the skull. GRIN lens and headbars were secured to the skull using Loctite adhesive and further secured using Metabond. Recordings began at least 4 weeks after GRIN lens implant.

#### Rabies tracing

For rabies-mediated monosynaptic retrograde tracing, 90–120nl of a rabies ‘helper’ vector mix were injected into the LS. Two weeks later, 150–200nl CVS-N2c was injected into the LS. For monosynaptic input tracing from *Crhr2* subpopulations, rabies ‘helpers’ comprised of an AAV-hSyn-CV-TVA(mCherry)-W3SL and AAV-hSyn-CV-N2cG:HA-W3SL (plasmids provided by Lindsay Schwarz; generated by BCH viral core) mix and were followed by a CVS-N2c-h2b-GFP (UNC Viral Vector Core) injection. Brains were perfused 10 days following injection for Serial Two-Photon Microscopy (STPT) imaging (see *Serial Two Photon Microscopy Methods*).

#### Rabies-assisted fiber photometry

For rabies-assisted fiber photometry of LS^*Crhr2*^ inputs, rabies ‘helpers’ comprised of an AAV-CAG(del)-TVA:HA-FLEx(loxP) (Custom plasmid; BCH Viral Core) and AAV-CAG(del)-N2cG:YFP-FLEx(loxP) mix (Custom plasmid; BCH Viral Core). In the same surgery, ‘helper’ virus mix and AAV-CAG-GCaMP8m-FLEx(FRT) (Custom plasmid; BCH Viral Core) were injected in the LS and at the site for recording bulk calcium activity at the input site of interest, respectively. 14–21 days later, we injected EnVA-CVS-N2c-Flp-tdTomato (BCH Viral Core) in the LS and implanted with a 400um fiber optic at the recording site. 2 anchor screws were secured to the skull. Fiber optic ferrule and headbars were secured to the skull using Loctite adhesive and further secured using Metabond. Recordings began no sooner than 10 days after N2c-Flp-tdTomato injection, and mice were perfused no later than 24 days post-injection to ensure minimal cell-death^51^.

#### In vivo optogenetics

For *in vivo* optogenetic manipulations of the LS^*Crhr2*^ population, AAV5-CAG(del)-Archaerhodopsin3:eYFP-FLEx(loxP) (custom plasmid; BCH Viral Core) was injected bilaterally in the LS. 14 days later, we implanted a 200um fiber optic in each hemisphere for bilateral LS inhibition. Anchor screws were secured to the skull. Fiber optic ferrule and headbars were secured to the skull using Loctite adhesive and further secured using Metabond. Behavioral experiments began 7–10 days after the implant, and mice were perfused no later than 4 weeks post-injection for histological verification.

### Behavior

All behavioral tests were conducted during the dark phases beginning no sooner than one hour after lights off. Mice were habituated to a room adjacent to the behavior rooms for at least 1 hour prior to behavioral testing. All mice used for optogenetic, fiber photometry, or microendoscopic recordings were habituated to cable tethering for 3 days before experiments started.

#### Signaled active avoidance

Signaled active avoidance (AA) was performed using a soundproofed shuttle box apparatus (Med Associates) where the two chambers of the apparatus were separated by two adjacent walls rather than a door to allow the passage of tethered fibers and wiring on the mouse’s head. The AA protocol consisted of multiple sessions, 30 trials per session, 1 session per day, and number of sessions depended on learning rate of each individual animal^65^. The training protocol comprised of an initial 10min baseline/habituation period, followed by random inter-trial intervals (ITIs, 25 to 40s) followed by a 10s, 75dB, 5KHz tone (conditioned stimulus, CS), immediately followed by a 0.4mA footshock (unconditioned stimulus, US) delivered through a metallic floor grid. The footshock was set to 15 s and was terminated when the animal shuttled to the opposite chamber. If the animal shuttled during CS delivery, the US was precluded, and the CS was terminated upon shuttle crossing. It is important to note that although footshock was set to a maximum of 15 s, animals seldom endured the shock for more than 2 s. Custom MedPC code output CS presentation and termination times (representative of success if <10-s or failure trials if >10-s for each CS). Alignment of stimulus presentation, behavior, and neural recordings were determined based on output TTL trigger times from MedPC to TDT Synapse software.

To calculate speed and positional dynamics, we used DeepLabCut (DLC) to track nose, center, and tailbase points of each animal across all AA sessions. We trained a custom DLC model using labeled video frames captured from the AA setup, and extracted the pose estimations for further analysis. We converted the raw pixel coordinates into real-world centimeters using a calibrated scale factor given the dimensions of the AA shuttle box. For each tracked body point (nose, center, tailbase), we computed X, Y, and combined XY coordinates, and a smoothed velocity using a Gaussian-filtered derivative over a 15-frame (0.5s) window, and derived absolute speed from the XY trajectories. Av-run onsets were defined as the timepoint at which speed reached 5 cm/s.

For analysis of neural recording experiments (microendoscopic and fiber photometry), we excluded trials where animals successfully shuttled in response to the CS prior to 4-seconds in order to distinguish signals related to CS versus Av-runs. Recordings were performed throughout training until “testing” (at least 33% successful trials).

For optogenetic inhibition experiments using Archaerhodopsin (Fig. 1c–d, **Extended Data Fig. 2b-j**), animals were tethered each day of training and trained until they met a separate criterion of 50% successful trials. The day at which criterion was reached marked the “pre”-stimulation day. Inhibition occurred the day after criterion was reached with delivery of a constant 535nm DPSS laser (Shanghai Laser Optic Century) at ~5mW. Laser illumination commenced 0.5 seconds prior to CS onset and was ramped down over 1 second upon shuttling or shock delivery. The following day (i.e. post-stim), mice were tested once again in the absence of laser delivery.

#### Open field test and auditory stimulus presentations

Mice were tested in an open field test (OFT) arena measuring 50 × 50 × 30 cm, constructed from white 1/8” ABS plastic. Animals were placed in a corner of the arena at the start of each assay. All sessions were video recorded using EthoVision (Noldus) for subsequent behavioral tracking. Pose estimation was performed using DLC, manually labeling keypoints along the nose, ears, neck, back, flanks, and tail for training a DLC model with a subset of videos of the OFT. Spontaneous behavior was first recorded for 15 minutes and segmented into behavioral syllables using keypoint-MoSeq^66^ for unsupervised classification of fine-scale motor sequences. Syllable usage and transition data were obtained using built-in functions in the Keypoint-MoSeq package. For stimulus responsivity assays, animals were presented with a series of 5-second auditory stimuli at 75 dB, delivered with 30–40 s inter-trial intervals (ITIs). Animals received 10 repetitions of a neutral 2 kHz tone, 5KHz tone, ultrasonic sweeps (USS), or white noise (WN). USS comprised of repeated 100 ms frequency sweeps from 17 to 20 kHz sweeps, typical of rat distress vocalizations^67^ that elicit defensive behaviors in mice^68^. WN presentations have also been shown to innately elicit defensive behaviors in mice^69^. Stimulus timing and ITIs were controlled and logged via a custom MATLAB script, and aligned with behavioral data using timestamps. Speed and position was calculated from extracted DLC kinematic trajectories around each stimulus presentation. Speed traces were normalized to stimulus onset and analyzed using parameters optimized to detect freezing (speed < 0.25 cm/s for ≥ 0.5 seconds) and flight (speed > 50 cm/s).

#### Pavlovian threat conditioning

Pavlovian threat conditioning (PTC) (Extended Data Fig. 2k-o) was conducted in a Med Associates operant chamber equipped with a speaker, shock grid, and ambient lighting controls. Animals underwent a four-day protocol across distinct phases. On Day 0 (pre-exposure), mice were placed in the conditioning chamber and presented with five auditory CS (conditioned stimulus) tones (7.5 kHz, 80 dB, 10 s duration), following a 3-minute baseline period. On Day 1 (conditioning), mice received ten CS–US (unconditioned stimulus) pairings, where the auditory CS co-terminated with a 1-second, 0.4 mA foot-shock. Trials were separated by pseudorandom inter-trial intervals (ITIs). On Day 2 (tone test), mice were re-exposed to the auditory CS for 20 trials without shock; green laser light (532 nm, continuous, ~5 mW at fiber tip, 1-second ramp down) was delivered throughout the CS period. On Day 3 (extinction), mice again received 20 CS-only trials without laser. All sessions were video recorded and analyzed using Video Freeze software (Med Associates Inc.) to quantify freezing behavior, defined as the absence of movement (below a motion index threshold) for ≥0.5 seconds. Freezing was computed as the percentage of time spent immobile during each CS presentation.

#### Head-fixed pupillometry and stimulus presentations

All head-fixed experiments were conducted in a sound-attenuating behavioral chamber (Med Associates) equipped for head-fixed assays. Mice were positioned on a freely rotating running wheel outfitted with a rotary encoder to track locomotion. Continuous illumination was maintained within the chamber, and pupil dynamics were recorded using an infrared-sensitive FLIR Flea3 USB 3.0 camera operating at 30 frames per second. Real-time tracking of pupil diameter was performed using a custom workflow built in Bonsai 2.3 (OpenCV), which applied image thresholding and binarization to identify the pupil and extract its largest diameter on each frame. A brief dark period was used to determine each animal’s maximal pupil size prior to testing.

For experiments involving physiological monitoring, animals were fitted with a pulse oximeter collar (MouseOx Plus, Starr Life Sciences) to record heart rate, respiratory rate, and oxygen saturation. Blink artifacts were identified and removed offline using custom MATLAB code. For neural recording experiments (**Extended Data Fig. 7e-i**), calcium imaging occured while head. On Day 1 (training), mice received 10 CS+ trials consisting of a 5 kHz tone (80 dB) co-terminating with a 0.2 mA tail shock (US) and 10 CS- neutral trials consisting of a 12 kHz tone (80 dB). On Day 2 (test), mice received 35 CS+ presentations with shock (to prevent extinction) and 35 CS- presentations, each delivered at five sound intensities (45,60,70,80,90,100 dB, 5 trials each). Locomotor speed and pupil size were aligned to stimulus onset and analyzed across intensities to quantify behavioral and physiological responses to sound salience. Neutral CS- data not shown.

For optogenetic inhibition experiments using Archaerhodopsin (Arch3; **Extended Data Fig. 2p–z**), mice were head-fixed on the same apparatus. A MouseOx Plus pulse oximeter collar (Starr Life Sciences) was used to record heart rate, respiration, and oxygen saturation, while pupil size was tracked as above. Following a 10-minute baseline acclimation, animals underwent Pavlovian threat conditioning (PTC) with 5 CS–US pairings (0.2 mA tail shock co-terminating with the 10 s CS+ tone). After conditioning, animals received 10 CS+ tone presentations during which Arch3-expressing mice received bilateral laser stimulation (532 nm, 10 s continuous, 1-second ramp down, ~5 mW at fiber tip). Behavioral and physiological parameters were analyzed and assessed using custom MATLAB scripts.

### Microendoscopic imaging

#### Data acquisition

Calcium imaging of Crhr2 neuronal populations and subpopulations was performed using either the Doric Snap-In Imaging Cannula system or the Inscopix nVoke platform with integrated ProView GRIN lenses. Doric-based recordings were acquired continuously at a frame rate of 5 Hz throughout the entire behavioral session. Inscopix-based recordings were collected at a frame rate of 20 Hz and restricted to specific imaging epochs, consisting of an initial 3-minute baseline recording and discrete trial segments spanning from 5 seconds before conditioned stimulus (CS) onset to 10 seconds after CS offset.

#### Image registration and signal extraction

Raw fluorescence movies were spatially aligned and motion-corrected using either the Normcorre-based ExTRACT pipeline^70^ or Suite2p^71^. Neuronal regions of interest (ROIs) were defined based on spatial footprints generated by these pipelines, and corresponding temporal calcium signals were extracted. These spatial footprints provided robust neuron identification and allowed consistent tracking across trials and sessions. After extraction, individual neuron calcium traces underwent preprocessing steps before alignment to behavioral epochs and subsequent analyses.

#### Preprocessing

All acquired calcium imaging data were preprocessed to remove neuropil contamination by subtracting a neuropil signal scaled by a factor of 0.7 (i.e., F – 0.7 × F_neuropil) from each neuron’s raw fluorescence trace. Fluorescence traces were normalized using a sliding-window method, in which a moving baseline fluorescence value (F_0_) was calculated as the 10th percentile of fluorescence intensities within each consecutive, overlapping 25-second window along the recording. Specifically, for each neuron, a dynamic baseline fluorescence (F_0_) was calculated as the 10th percentile of fluorescence intensities within a moving trial window. The resulting ΔF/F traces were concatenated across trials within a session. After concatenation, z-score normalization was performed across the entire session-length trace for each neuron individually. Dropped frames were identified as artifacts and were linearly interpolated prior to further analyses.

#### ROC analysis

Receiver operating characteristic (ROC) analysis^72^ was used to identify neurons that were significantly modulated during each task variable assessed during active avoidance. To do so, we generated a ‘baseline’ distribution of fluorescent values for each neuron and trial for each task variable (i.e. CS, av-run, and shock: 2 s prior to stimulus onset; pre-av-run: 3 to 2 s prior to av-run). This distribution was then compared to each timepoint in a trial and the area under the ROC curve (auROC) was calculated. The auROC value is in estimate that describes how discriminable two distributions are. For example, if no timepoints in the ‘task variable’ period overlap with the baseline distribution, auROC will return an estimate of 1 (increase in activity compared to baseline) or 0 if all values are lower (decrease in activity compared to baseline), while an auROC estimate of 0.5 describes the two distributions as indistinguishable. Average auROC estimates for each neuron and task variable were calculated by averaging across trials (i.e. CS, av-run, and shock: 2 s following stimulus onset; pre-av-run: 2 s prior to av-run). These values were then compared to a null distribution of auROC estimates generated from ROC curves constructed from trials composed of randomly permuted calcium signals (i.e. traces for each trial generated after circularly permuting fluorescent signal in a session 1000 times). A neuron was considered ‘significantly modulated’ if the estimated auROC was outside of the 95% confidence interval (a = 0.05) – activated if auROC > 97th percentile and suppressed if auROC < 2.5th percentile.

#### Population dynamics

To understand population-level neural activity dynamics, we applied a targeted dimensionality reduction technique, principal components analysis (PCA), that maximizes the variance in the data. We assumed that all n neurons from different mice and trials could have been recorded simultaneously and thus were pooled together to make a trial-averaged matrix X (n x t dimensions) aligned to either the onset of the conditioned stimulus (CS-onset) or the avoidance run (Av-run), where each row represents a single neuron and each column represents a single timepoint, for a single global PCA. To capture the variance in the data, we built a matrix containing trial-averaged activity for n units during both success and failure trials by concatenating the two trial types for each neuron to form a n x 2t matrix. PCA was then performed on this concatenated matrix, yielding a set of orthogonal principal components (PCs) that best captured the population variance across time. Time windows used for dimensionality reduction were −2 to +3 seconds relative to CS onset and −3 to +3 seconds relative to Av-run. All neural data were normalized and mean-centered prior to analysis. To examine trial-to-trial differences, we projected single-trial neural activity from each condition onto the trial-averaged PCA subspace. This yielded low-dimensional trajectories for individual trials that could be visualized and compared in terms of their temporal evolution in PC space. For visualization, the first three PCs were used to reconstruct population trajectories (Fig. 1k and n, **Supplementary Videos 1 and 2**). Due to variability in trial availability across animals, the number of trials used per condition for each neuron was limited to the lowest number available across the dataset. To quantify differences in neural trajectories between trial types, we computed the Mahalanobis distance between success and failure trajectories in the reduced PC space. For each iteration, a random subset of neurons was sampled, and PCA was applied to trial-averaged data to extract the number of PCs required to explain 80% of the variance. These PCs were then used to project single-trial activity, and Mahalanobis distances were computed at each time point between success and failure trials, as well as between each trial type and its own baseline period (defined as −2 to 0 seconds for CS-onset, and −3 to −2 seconds for Av-run). This process was repeated across 100 bootstrapped iterations to ensure reliability. The resulting time series of Mahalanobis distances were z-scored to baseline, and plotted across time. Statistical significance of separation between trial types was assessed using pointwise permutation tests across iterations. At each time point, we calculated the proportion of iterations where the Mahalanobis distance between success and failure trials was less than a conservative threshold (z < 1.65), and considered time points significant if this occurred in fewer than 5% of iterations^29^.

#### Population decoding

To determine how well trial-type (i.e. success or failure) during CS-onset and av-runs in the active avoidance task was captured in LS Crhr2 population activity at the level of single-trials, we used linear-kernel support vector machine (SVM) classifiers using the fitcsvm MATLAB function. In order to generate pseudo-population vectors for classification, we reconstructed single-trial neural activity for each neuron using coefficients for the top PCs explaining 80% of the variance in the global PCA (see *Population dynamics Methods*). First, in order to assess whether the number of neurons used for classification affected decoding accuracy, we randomly sampled 1, 10, 50, 100, 200, 300, 400, 500, or 1000 neurons for pseudo-population vector construction. Here, only the CS-period (0 to 2-sec after CS onset) or pre-av-run period (−1.4 to 0-sec preceding av-run) were used for classification by concatenating those time periods for all neurons in each trial, resulting in a 1 x (n x t) pseudo-population vector for each trial, where n is number of neurons and t is number of frames for a trial (i.e. number of 200-ms time bins). In order to account for variability in accuracy, we performed 100 bootstrap iterations with randomly selected trials for each randomly-sampled neuron to obtain a pseudo-population vector for each trial. Owing to the low number of trials available (see *Signaled active avoidance Methods*), ‘leave-one-out’ stratified cross-validation was performed (i.e. one trial held out from each class). We defined decoding accuracy of each classifier as the percentage of correctly classified trials in the cross-validation procedure.

For time-varying decoders, instantaneous pseudo-population vectors were constructed for each data point (i.e. 200-ms or 50-ms time bins for 5Hz or 20Hz recordings, respectively) from single-trial neural activity reconstructed using the PC coefficients (>80% variance explained) for each neuron in the global PCA, as described above. Again, to account for variability in accuracy, we performed 100 bootstrap iterations with randomly selected trials to generate t number of 1 x n pseudo-population vectors for each trial and timepoints. Since decoding accuracy depends on the number of neurons included in the training set (**Extended Data Fig. 3g-h**), we randomly sampled 200 neurons to generate the pseudo-population for each trial-type and bootstrap run to generate the instantaneous pseudo-population vectors used for classification.

To evaluate decoding accuracies due to variability, we randomly shuffled the class labels for each trial 1000 times and trained and assessed the classifiers in a manner identical to that with the real data. The decoding accuracies obtained with shuffled labels were used to generate a null distribution of the decoding accuracies that would occur by chance. Decoding accuracies obtained from shuffled class labels were then directly compared to that obtained from real data.

#### Functional clustering of neuronal populations

To identify functionally distinct subgroups among recorded neurons (Fig. 2, **Extended Data Fig. 6 and 11h-l**), we performed unsupervised clustering on population activity patterns using a principal components analysis (PCA)–based dimensionality reduction followed by *k*-means clustering. Trial-averaged activity for each neuron across four behaviorally defined time windows (CS-success, CS-failure, shock, and avoidance run) was concatenated to form a neuron × time matrix. PCA was applied to the concatenated matrix to reduce dimensionality while preserving variance in the data. The number of principal components retained was determined by the number required to explain at least 80% of the total variance.

We then applied k-means clustering to the PCA-reduced data, systematically varying the number of clusters from 1 to 20 and evaluating within-cluster distances to identify the inflection point in the elbow curve. Based on this analysis, we selected 10 as the optimal number of clusters. The silhouette score was used to validate the clustering solution, and all clustering was performed with 5 replicates to ensure robustness. Each neuron was assigned to one of the 10 clusters based on proximity in PCA space. The neurons within each cluster were sorted by similarity in trial-averaged response profiles, and the activity traces were normalized on a per-neuron basis to a [0,1] range to facilitate visualization. For display, cluster-ordered heatmaps were generated to show the average response of neurons in each cluster across all behavioral epochs.

#### Spatial organization of functional neuronal clusters

To assess the spatial organization of functional neuronal clusters, neuronal positions were grouped according to cluster identity. We first prepared and binned the data by mapping neuronal positions along the anteroposterior (AP), mediolateral (ML), and dorsoventral (DV) axes. The neuronal positions were divided into eight bins for each axis using histcounts in MATLAB. For each bin, we calculated the mean anatomical position value and then normalized these values by the total sum of the mean anatomical position across all bins to obtain relative concentrations. To assess the statistical significance of these relative concentrations, we randomly shuffled the coefficient data 10,000 times to generate a null distribution. For each shuffle iteration, relative concentrations were recalculated. From this shuffled data, 95% confidence intervals were calculated, and the standard deviation of the actual relative concentrations was compared to the standard deviations of the shuffled data. P-values were then computed by comparing the standard deviations of the actual data to those of the shuffled distributions (Extended Data Fig. 8d-f).

#### Coding direction

To analyze the neural activity differences between success and failure trials, we computed the coding direction (CD) and projected the neural activity data along this direction as performed previously^33^. The coding direction is a vector in the n-dimensional space of neural activity that best separates the neural responses between the two trial types. For CS, we extracted the data for the time window from 0 to 3 seconds following the CS onset for both success and failure matrices (Fig. 2, **Extended Data Fig. 5**). The neural activity was averaged over the specified time window for both success and failure trials. This produced two n-dimensional vectors representing the mean neural activity for each trial type. For success trials, the average neural activity vector is denoted as x(t)^success^, and for failure trials, it is denoted as x(t)^failure^. We computed the difference vector by subtracting the average neural activity vector of the failure trials from the average neural activity vector of the success trials: d = x(t)^success^ – x(t)^failure^. This difference vector captures the direction in the n-dimensional space along which the neural activity for success and failure trials is maximally different. To convert the difference vector into a unit vector (the coding direction, CD), we divided it by its magnitude. The projection of the neural activity data x onto the CD vector was computed by taking the dot product of the CD vector with the neural activity matrix: CDTx. This resulted in a time series of projections that distinguish between success and failure trials. By construction, the projection values are positive during success trials and negative during failure trials. To characterize additional variance in the population response that was not captured by the CD, we computed PCs from the residual trial-averaged activity orthogonal to the CD. Neural activity from each trial was projected onto these orthogonal PCs to obtain low-dimensional trajectories reflecting variance not aligned with task outcome. The top PCs, which captured the majority of the remaining variance (up to 85%), were analyzed separately for each trial type. These projections revealed structure in the neural dynamics independent of cue-outcome encoding, providing a complementary view of the population activity space.

#### Spatial organization of PCA or CD coefficients

To analyze the spatial organization of PCA or CD coefficients, we first prepared and binned the data by mapping neuronal positions along the anteroposterior (AP), mediolateral (ML), and dorsoventral (DV) axes. The neuronal positions were divided into eight bins for each axis using histcounts in MATLAB. For each bin, we calculated the mean coefficient value and then normalized these values by the total sum of the mean coefficients across all bins to obtain relative concentrations. To assess the statistical significance of these relative concentrations, we randomly shuffled the coefficient data 10,000 times to generate a null distribution. For each shuffle iteration, relative concentrations were recalculated. From this shuffled data, 95% confidence intervals were calculated, and the standard deviation of the actual relative concentrations was compared to the standard deviations of the shuffled data. P-values were then computed by comparing the standard deviations of the actual data to those of the shuffled distributions and corrected using the Hom-Bonferroni correction for multiple comparisons (Fig 4e,k and 5e, j).

#### Generalized Linear Model (GLM)

Neural activity was modeled using a generalized linear model (GLM) to quantitatively relate behavioral events to concurrently recorded calcium signals (**Extended Data Fig. 3b-e and 11o-t**). Behavioral events – such as cue onsets for both successes and failures, shock deliveries, and Av-run onset – were organized into binary time-series arrays. Neural activity from multiple subjects was similarly segmented into trial-by-trial matrices. All analyses were performed in MATLAB (MathWorks Inc.) using ordinary least squares (OLS) regression and to minimize overfitting. To account for the slow kinetics of the calcium indicator, each binary behavioral event was convolved with a GCaMP kernel. This kernel was defined with a rise time of 0.15 s, a decay constant of 0.8 s, and a total duration of 1.5 s. The convolution process transformed the discrete event markers into continuous time courses that more closely resembled the fluorescence dynamics of the calcium indicator. In addition, to adjust for potential delays between the onset of a behavioral event and the corresponding neural response, the convolved traces were shifted temporally. Predictors were shifted by up to 2 s. Cue-onset (success and failure), shock onset, and Av-run onset were shifted in the forward directions while anticipatory Av-runs were shifted in the backward directions, effectively aligning the behavioral and neural signals.

For each trial, the convolved and shifted predictor time courses were concatenated to form a comprehensive design matrix. The GLM was applied on a per-cell basis using OLS regression. For each neuron, the design matrix was used to predict the corresponding calcium signal recorded during the trial. To assess model performance, leave-one-out cross-validation (LOO-CV) was employed. In LOO-CV, each trial was sequentially held out as a test set while the model was fit on the remaining trials. The coefficient of determination (R^2^) was computed between the predicted and observed neural activity, and mean squared error (MSE) was calculated for each fold.

To quantify the influence of individual behavioral events on neuronal activity, nested model comparisons were performed. For each behavioral variable, predictors corresponding to that variable were either zeroed (without re-estimating the model) to simulate its omission. The resulting drop in R^2^, normalized by the full model’s R^2^, was computed to yield a relative contribution metric reflecting the percentage impact of that variable on the neural response. The associated F-statistics from these comparisons provided an additional index for evaluating the significance of behavioral contributions. The final analysis yielded regression weights (β coefficients), full-model performance metrics (R^2^), relative contribution percentages, F-statistics, and trial-specific predictions of neural activity. These results were organized into structured tables indexed by subject, providing quantitative results for interpreting how distinct behavioral events modulate neuronal dynamics.

### Fiber photometry

#### Data acquisition

Optical fibers (1 m length, 400 μm core, 0.48 NA; Doric Lenses) were connected to implanted fibers using zirconia or bronze sleeves (Doric Lenses). Excitation and emission light were transmitted through a fluorescence minicube (FMC6_AE(405)_E1(465–480)_F1(500–540)_E2(555–570)_F2(580–680)_S; Doric Lenses). The excitation light (~40 μW) was delivered by 405 nm and 465 nm LEDs (Doric), modulated at 531 Hz or 211 Hz, using an RZ5P or RZ10 fiber photometry processor (Tucker-Davis Technologies, TDT) running Synapse software. Emission light was collected via fluorescence photodetector heads integrated into the minicube (Doric Lenses). Signals were demodulated, digitized at 1 kHz, and recorded through the processor. Transistor-transistor logic (TTL) timestamps were used to synchronize the signal with video frames and experimental stimuli. Photometry recordings were conducted at least 4 weeks after viral vector injection and 10 days after implantation.

#### Data analysis

Both the 465 nm and 405 nm signals were downsampled to 30 Hz, matching the sampling rate of the behavioral video recordings. The signals were then smoothed and detrended using a linear regression model. The best-fit 405 nm signal was subtracted from the 465 nm signal, and the result was divided by the fit to obtain the relative fluorescence change, ΔF/F, expressed as a percentage. For active avoidance, trials were spliced, concatenated, and Z-scored for normalization. Traces from all other experiments were Z-scored before splicing. The ΔF/F time course was plotted, and all fluorescence traces were visually inspected for quality control. Any traces with significant motion artifacts, detected via the 405 nm channel, were excluded from further analysis.

#### Generalized Linear Model (GLM)

Neural activity from each afferent was modeled using a generalized linear model (GLM) to quantitatively relate behavioral events to concurrently recorded calcium signals in a manner identical to that of the microendoscopic imaging of single neurons (see Microendoscopic imaging Methods). Due to these data representing bulk calcium signals as opposed to single neuron activity, the time-varying bulk signal from each mouse was treated as a “single neuron” (Fig 5h–j and **Extended Data Fig. 15j-p**).

#### Decoder Analysis

SVM decoders were trained and tested in a similar method to that described in Population Decoding in the Microendoscopic Imaging Methods, Here, only the CS-period (0 to 2-sec after CS onset) or pre-av-run period (−1.4 to 0-sec preceding av-run) were used for classification by concatenating those time periods for all neurons in each trial, resulting in a 1 x (n x t) vector for each trial, where n is number of mice recorded or that region and t is number of frames for a trial. In order to account for variability in accuracy, we performed 100 bootstrap iterations with randomly selected trials for each randomly-sampled mouse to obtain a pseudo-population vector for each trial. Owing to the low number of trials available (see *Signaled active avoidance Methods*), ‘leave-one-out’ stratified cross-validation was performed (i.e. one trial held out from each class). We defined decoding accuracy of each classifier as the percentage of correctly classified trials in the cross-validation procedure (Fig. 5k–m).

#### Immunohistochemistry and Histology

Mice were anesthetized with a ketamine and xylazine dilution, and sequentially perfused with saline and phosphate buffered saline (PBS) containing 4% paraformaldehyde (PFA). Brains were removed and incubated in PBS containing 4% PFA overnight on a rocking platform at 4°C. For vibratome sectioning, brains were sectioned at 75–100 μm thickness. For microtome sectioning, brains were cryoprotected using 30% sucrose in PBS for at least 24 hours and sectioned at 50um thickness. Fluorescent imaging was performed a whole slide scanning microscope with 719 a 10X objective (Olympus VS120 slide scanners), or acquired with an upright confocal microscope (Zeiss LSM 800) with 720 a 10X objective (Plan-Apochromat 10x/0.45 M27) or a 20X objective (Plan-Apochromat 20x/0.8 M27). Mice in which optical fiber placements were not positioned over the respective targets were discarded from the analysis. Stitching of tiled images was performed using Zeiss Zen software and processed using ImageJ Fiji software.

### Single nuclei RNA sequencing (snRNAseq)

#### Nuclei suspension preparation and library construction

Brain samples were harvested from *Crhr2*-IRES-Cre; LSL-H2B-GFP mice at postnatal days 50–53 and immediately placed in ice-cold Homogenization Buffer containing 0.25 M sucrose, 25 mM KCl, 5 mM MgCl_2_, 20 mM Tricine-KOH (pH ~7.8), 1 mM DTT, 0.15 mM spermine, and 0.5 mM spermidine. Lateral septum tissue was microdissected and transferred into a 2 ml Dounce homogenizer filled HB buffer supplemented with 0.25% IGEPAL-CA630 and 0.2 U/μl RNasin.

Tissue homogenization was performed with 8–10 strokes of the pestle A. After a 5-minute incubation on ice, the homogenate received additional 9–10 strokes with the pestle B. The resulting suspension was passed through a 30 μm filter into a 15 ml conical tube, then centrifuged at 500 × g for 5 minutes at 4 °C (swinging-bucket rotor). The nuclear pellet was gently resuspended in 1XPBS containing 1% BSA and 0.2 U/μl RNasin, filtered through a 40 μm strainer, and stained with DAPI. GFP-positive nuclei were isolated on a Sony MA900 sorter using a 70 μm nozzle (see Supplemental Methods). Sorted nuclei were collected into chilled 0.2 ml PCR tubes and counted with an INCYTO C-Chip hemocytometer. snRNA-seq libraries were prepared with the 10x Genomics Chromium Single Cell 3′ v3.1 kit according to the manufacturer’s guidelines and subsequently sequenced on an Illumina NovaSeq 6000.

#### snRNA-seq data processing

CellRanger (v6.1.2, 10x Genomics) was used with default parameters to map snRNA-seq data to the Mus musculus reference genome (mm10) provided by 10x Genomics. Filtered gene-barcode matrices were imported into R using the Seurat package (v4.0.5), where initial quality control was performed to exclude low-quality nuclei and potential doublets. Nuclei with fewer than 1,500 detected genes or more than 6,500 detected genes were removed, and mitochondrial gene content was quantified and regressed out during normalization. Gene expression was normalized and variance-stabilized using SCTransform with the glmGamPoi method, regressing out mitochondrial content.

Dimensionality reduction was performed using principal component analysis (PCA), with the top 200 PCs retained for downstream clustering. Shared nearest neighbor (SNN) graphs were constructed using the top principal components, and unsupervised clustering was performed using the Louvain algorithm across a range of resolutions to identify subclusters. Clusters were visualized using Uniform Manifold Approximation and Projection (UMAP), and marker genes were identified for each cluster using Wilcoxon rank-sum tests implemented in Seurat’s FindAllMarkers function. To focus specifically on LS populations, clusters expressing LS-enriched markers (e.g., *Trpc4, Prdm16, Crhr2*) were manually selected and extracted for reanalysis. These LS-enriched clusters were further filtered based on expression of anatomical markers and subclustering performance, yielding a high-confidence set of LS *Crhr2*-expressing cells. Marker gene expression for LS clusters and subclusters was visualized using *FeaturePlot, DotPlot*, and *DoHeatmap* functions.

For advanced visualization and downstream quantitative analyses, the final filtered Seurat object was converted to AnnData format and imported into Python. All figures and marker visualizations were subsequently generated using Scanpy (v1.9.3), leveraging functions for dimensionality reduction, clustering, and feature plotting in a consistent cross-platform workflow.

#### Crhr2 neuron snRNA-seq integration with LS-wide MERFISH

To compare the transcriptomic profile of LS *Crhr2* neuronal subtypes across modalities, we integrated our single-nucleus RNA-seq (snRNA-seq) dataset with publicly available MERFISH data^40^. Preprocessed and filtered AnnData objects were normalized using total-count normalization (target sum = 10,000) followed by log transformation. To enable direct comparison and alignment of shared transcriptional features, we applied Scanorama, a batch-correction and integration method designed for multi-dataset single-cell analyses. Scanorama was used to identify shared gene expression structure across datasets and return a corrected joint embedding.

The integrated dataset was used to compute a shared nearest neighbor graph based on the top 40 Scanorama components, followed by dimensionality reduction with UMAP and clustering using the Leiden algorithm. To relate cell types identified in snRNA-seq with those in MERFISH, we calculated Pearson correlations between average gene expression vectors of snRNA-seq clusters (annotated by SCT_snn_res.1.2) and MERFISH-derived Leiden clusters. This correlation matrix was used to identify best-matching pairs of clusters across the two datasets.

To further refine comparisons, a subset of MERFISH clusters showing the strongest correspondence to snRNA-seq clusters was selected for reclustering at higher resolution. The resulting high-resolution clusters were assigned putative molecular identities by cross-referencing correlated snRNA-seq clusters and differentially expressed genes. Subtypes were annotated with marker-defined labels: *Glp1r, Chat, Lhx2, Met, Tshz2, Foxp2*, *and Calcr*, and validated via spatial expression patterns in the original MERFISH dataset.

To visualize the anatomical distribution of these subtypes, integrated annotations were mapped back onto the MERFISH spatial coordinates, and individual subtype distributions were visualized using scanpy.pl.spatial. Final correlation matrices comparing integrated Leiden clusters (subgroups) with subtype assignments were used to confirm correspondence across datasets. The annotated and integrated datasets were then used for downstream visualization and spatial mapping.

#### Crhr2 neuron snRNA-seq integration with LS-wide snRNAseq

To compare LS Crhr2 neuronal subtypes identified in our snRNAseq dataset to previously identified transcriptionally defined clusters across the LS^40^, we integrated our snRNA-seq dataset (referred to as DBM) with the LS-wide snRNA-seq dataset from Reid et al. (2024) collected from FAC sorting GFP positive and negative cells from Nkx2.1-Cre mice (referred to as CMR). Both datasets were normalized using total-count normalization (target sum = 10,000) followed by log-transformation. Integration was performed using Scanorama, which corrects batch effects by aligning shared transcriptomic structure across datasets. The corrected low-dimensional embeddings returned by Scanorama were used to construct a joint neighbor graph and compute a UMAP embedding.

The integrated data were clustered using the Leiden algorithm, and cluster assignments from each dataset (e.g., SCT_snn_res.1.2 from the DBM data and subgroups from the CMR data) were overlaid onto the integrated UMAP to assess convergence of biological signal across datasets. To quantitatively assess concordance between annotated clusters, we computed Pearson correlation coefficients between the average gene expression profiles of clusters across datasets. Expression values were grouped by cluster, averaged across all cells, and the resulting correlation matrix was visualized as a heatmap to reveal transcriptional similarity across cluster pairs.

To further investigate specific shared subtypes, we subsetted individual clusters from the CMR dataset (e.g., LSN 11, LSN 12, LSN 5, LSN 1, LSN 3, LSN 8), re-clustered each region independently at higher resolution, and repeated the correlation analysis against DBMclusters. This allowed for detailed mapping of individual Crhr2-expressing subpopulations—such as *Glp1r, Chat, Lhx2, Met, Tshz2, Foxp2*, and *Calcr* expressing types—onto the broader LS transcriptional landscape. Expression of *Crhr2* and other marker genes within these subclusters was visualized on UMAP projections to confirm molecular identity. Expression matrices and all resulting integrated annotations were retained for downstream analyses and visualization of subpopulation structure across datasets.

### Serial Two-Photon Tomography (STPT)

#### Data acquisition

Perfused mouse brains (see Immunohistochemistry and Histology Methods) were sectioned and imaged on a TissueCyte serial two-photon microscope (TissueVision Inc.) equipped with a 16×, 1.0 numerical aperture objective. Images were acquired at a resolution of 832 × 832 pixels per frame, with continuous automated acquisition across the entire brain volume. Following acquisition, raw image tiles were stitched using TissueVision’s proprietary stitching software to produce continuous volumetric reconstructions. Volumetric datasets were subsequently registered and aligned to the Allen Mouse Brain Common Coordinate Framework (CCFv3) using NeuroInfo (MBF Bioscience), which enables affine and deformable alignment of individual brain volumes to the reference atlas. Anatomical regions were auto matically segmented based on CCFv3 annotations. Quantification of signal across brain regions and hemispheres was performed using NeuroInfo’s integrated region-based analysis tools. Data were exported for further statistical analysis and visualization in MATLAB.

#### Comparisons Across Regions

To determine which brain regions differed between molecular subtypes across all proportions of inputs, we performed one-way ANOVA for each region independently. The dependent variable was the region-wise metric, and the independent variable was cell type. For each region, we extracted the F statistic, P value, and calculated effect size. False discovery rate (FDR) correction was applied across all regions using the Benjamini-Hochberg procedure to control for multiple comparisons. Where ANOVA revealed significant effects (FDR-adjusted P < 0.05), we performed post hoc multiple comparisons using Tukey’s Honest Significant Difference test. Group pairs with adjusted P < 0.05 were reported as significantly different (Fig. 4b). To compare the proportion of anatomical inputs within subregions (e.g., hypothalamus), we computed the proportion of total inputs assigned to each subregion for each animal or cell type. Proportions were then compared across groups using one-way ANOVA. When significant, pairwise comparisons were conducted with Tukey’s test, and FDR correction was applied across all subregions tested (Fig. 4f–g and **Extended Data Fig. 14c-g**).

#### Decoder Analysis

Data were organized as a structured dataset of cell proportion values across brain regions for each strain and individual mouse. Proportion data were extracted from each sample (e.g., for a specified subregion and hemisphere) and standardized using z-score normalization. To predict group membership (strain), we implemented a linear support vector machine (SVM) classifier using MATLAB’s fitceococ function. The classifier was trained on the standardized data, and its performance was evaluated using both bootstrapping and leave-one-out cross-validation (LOOCV). In the bootstrapping procedure, samples were resampled with replacement over 1,000 iterations, and the classifier was retrained for each iteration with performance tested on the complete dataset to generate an empirical accuracy distribution. LOOCV was employed by sequentially withholding each sample as a test case, yielding a single overall accuracy metric. Additionally, a permutation test was conducted by randomly shuffling the group labels for 1,000 iterations to construct a null distribution of accuracies, and a permutation p-value was computed as the proportion of shuffled iterations with accuracies equal to or exceeding that obtained with the true labels. Classifier performance was considered significant when accuracy exceeded the 95th percentile of the null distribution generated from shuffled data. Confusion matrices were computed for each model, and results were averaged across folds and iterations. (Fig. 4e).

#### Principal component analysis and similarity index

Principal component analysis (PCA) was performed on the same standardized dataset to reduce dimensionality and elucidate inter-group differences (Fig. 4c). The first three principal components (PCs) were retained, capturing the majority of variance in the cell proportion data. Three-dimensional scatter plots were generated to visualize the distribution of individual mice in PCA space, with points colored according to strain. Group means were computed for each subtype, and a combined visualization overlayed individual data points with the group mean markers to highlight both within-group variability and between-group differences. To quantitatively compare groups (i.e. subtype classes), pairwise statistical tests were conducted on the PCA scores. For each pair of groups, two-sample t-tests were applied to each principal component and the resulting p-values were combined using Fisher’s method. In a complementary Monte Carlo approach, all pairwise Euclidean distances between individual samples from different groups were computed and averaged to obtain a dissimilarity measure, which was then converted to a similarity index using the formula 1/(1 + distance). Permutation tests were performed by randomly reassigning samples to groups, thereby generating null distributions of average distances and corresponding p-values. Similarity and significance matrices were visualized, and hierarchical clustering was used to generate a dendrogram that illustrated the overall inter-group relationships based on the PCA-derived mean vectors (Fig. 4d).

## Supplementary Material

Supplementary Files

This is a list of supplementary files associated with this preprint. Click to download.

- LScrhr2manuscriptnatureDLBMextendeddata.pdf

## Data Availability

Sequencing data have been deposited at the Gene Expression Omnibus (GEO) under accession number GSE298976. All other data may be requested from the corresponding authors. Any custom code critical to the analysis of the data in the manuscript are available at https://github.com/dl-bm.

## References

[1] Risold P. Y. & Swanson L. W. Connections of the rat lateral septal complex. Brain Res Brain Res Rev 24, 115–195 (1997). 9385454 10.1016/s0165-0173(97)00009-x

[2] Besnard A. & Leroy F. Top-down regulation of motivated behaviors via lateral septum sub-circuits. Mol Psychiatry 27, 3119–3128 (2022). 35581296 10.1038/s41380-022-01599-3 PMC7613864

[3] Risold P. Y. & Swanson L. W. Chemoarchitecture of the rat lateral septal nucleus1Published on the World Wide Web on 2 June 1997.1. Brain Research Reviews 24, 91–113 (1997). 9385453 10.1016/s0165-0173(97)00008-8

[4] Franklin T. B., Saab B. J. & Mansuy I. M. Neural Mechanisms of Stress Resilience and Vulnerability. Neuron 75, 747–761 (2012). 22958817 10.1016/j.neuron.2012.08.016

[5] Henckens M. J. A. G., Deussing J. M. & Chen A. Region-specific roles of the corticotropin-releasing factor–urocortin system in stress. Nat Rev Neurosci 17, 636–651 (2016). 27586075 10.1038/nrn.2016.94

[6] Anthony T. E. Control of Stress-Induced Persistent Anxiety by an Extra-Amygdala Septohypothalamic Circuit. Cell 156, 522–536 (2014). 24485458 10.1016/j.cell.2013.12.040 PMC3982923

[7] Hashimoto M. Lateral septum modulates cortical state to tune responsivity to threat stimuli. Cell Reports 41, 111521 (2022). 36288710 10.1016/j.celrep.2022.111521 PMC9645245

[8] Sheehan T. P., Chambers R. A. & Russell D. S. Regulation of affect by the lateral septum: implications for neuropsychiatry. Brain Research Reviews 46, 71–117 (2004). 15297155 10.1016/j.brainresrev.2004.04.009

[9] Albert D. J. & Chew G. L. The septal forebrain and the inhibitory modulation of attack and defense in the rat. A review. Behavioral and Neural Biology 30, 357–388 (1980). 7013753 10.1016/s0163-1047(80)91247-9

[10] Wirtshafter H. S. & Wilson M. A. Lateral septum as a nexus for mood, motivation, and movement. Neuroscience & Biobehavioral Reviews 126, 544–559 (2021). 33848512 10.1016/j.neubiorev.2021.03.029

[11] Dai B. Experience-dependent dopamine modulation of male aggression. Nature 639, 430–437 (2025). 39843745 10.1038/s41586-024-08459-w PMC12136179

[12] Reyes N. S. de L. Corticotropin-releasing hormone signaling from prefrontal cortex to lateral septum suppresses interaction with familiar mice. Cell 186, 4152–4171.e31 (2023). 37669667 10.1016/j.cell.2023.08.010 PMC7615103

[13] Li L. Social trauma engages lateral septum circuitry to occlude social reward. Nature 613, 696–703 (2023). 36450985 10.1038/s41586-022-05484-5 PMC9876792

[14] Chen G. Cellular and circuit architecture of the lateral septum for reward processing. Neuron 112, 2783–2798.e9 (2024). 38959892 10.1016/j.neuron.2024.06.004

[15] Goode T. D. A dorsal hippocampus-prodynorphinergic dorsolateral septum-to-lateral hypothalamus circuit mediates contextual gating of feeding. bioRxiv 2024.08.02.606427 (2024) doi:10.1101/2024.08.02.606427. PMC13288035 41687615

[16] Besnard A. Dorsolateral septum somatostatin interneurons gate mobility to calibrate context-specific behavioral fear responses. Nat Neurosci 22, 436–446 (2019). 30718902 10.1038/s41593-018-0330-y PMC6387640

[17] Brady J. V. & Nauta W. J. H. Subcortical mechanisms in emotional behavior: affective changes following septal forebrain lesions in the albino rat. Journal of Comparative and Physiological Psychology 46, 339–346 (1953). 13109048 10.1037/h0059531

[18] Albert D. J. & Wong R. C. Hyperreactivity, muricide, and intraspecific aggression in the rat produced by infusion of local anesthetic into the lateral septum or surroundng areas. Journal of Comparative and Physiological Psychology 92, 1062–1073 (1978). 573285 10.1037/h0077524

[19] Ramirez J. M., Salas C. & Portavella M. Offense and Defense after Lateral Septal Lesions in Columba Livia. International Journal of Neuroscience 41, 241–250 (1988). 3182182 10.3109/00207458808990730

[20] Paxinos G. The septum: Neural systems involved in eating, drinking, irritability, muricide, copulation, and activity in rats. Journal of Comparative and Physiological Psychology 89, 1154–1168 (1975). 1238434 10.1037/h0077182

[21] Reis D. G., Scopinho A. A., Guimarães F. S., Corrêa F. M. A. & Resstel L. B. M. Involvement of the lateral septal area in the expression of fear conditioning to context. Learn. Mem. 17, 134–138 (2010). 20189957 10.1101/lm.1534710

[22] Calandreau L., Desgranges B., Jaffard R. & Desmedt A. Switching from contextual to tone fear conditioning and vice versa: The key role of the glutamatergic hippocampal-lateral septal neurotransmission. Learn. Mem. 17, 440–443 (2010). 20798266 10.1101/lm.1859810

[23] Radulovic J., Rühmann A., Liepold T. & Spiess J. Modulation of Learning and Anxiety by Corticotropin-Releasing Factor (CRF) and Stress: Differential Roles of CRF Receptors 1 and 2. J. Neurosci. 19, 5016–5025 (1999). 10366634 10.1523/JNEUROSCI.19-12-05016.1999 PMC6782638

[24] Henry B., Vale W. & Markou A. The Effect of Lateral Septum Corticotropin-Releasing Factor Receptor 2 Activation on Anxiety Is Modulated by Stress. J. Neurosci. 26, 9142–9152 (2006). 16957071 10.1523/JNEUROSCI.1494-06.2006 PMC6674509

[25] Todorovic C. Differential activation of CRF receptor subtypes removes stress-induced memory deficit and anxiety. European Journal of Neuroscience 25, 3385–3397 (2007). 17553007 10.1111/j.1460-9568.2007.05592.x

[26] Turner V. S., O’Sullivan R. O. & Kheirbek M. A. Linking external stimuli with internal drives: A role for the ventral hippocampus. Current Opinion in Neurobiology 76, 102590 (2022). 35753108 10.1016/j.conb.2022.102590 PMC9818033

[27] Biane J. S. Neural dynamics underlying associative learning in the dorsal and ventral hippocampus. Nat Neurosci 1–12 (2023) doi:10.1038/s41593-023-01296-6. PMC10448873 37012382

[28] Sternson S. M. Hypothalamic Survival Circuits: Blueprints for Purposive Behaviors. Neuron 77, 810–824 (2013). 23473313 10.1016/j.neuron.2013.02.018 PMC4306350

[29] Jercog D. Dynamical prefrontal population coding during defensive behaviours. Nature 595, 690–694 (2021). 34262175 10.1038/s41586-021-03726-6

[30] Heekeren H. R., Marrett S. & Ungerleider L. G. The neural systems that mediate human perceptual decision making. Nat Rev Neurosci 9, 467–479 (2008). 18464792 10.1038/nrn2374

[31] Evans D. A., Stempel A. V., Vale R. & Branco T. Cognitive Control of Escape Behaviour. Trends in Cognitive Sciences 23, 334–348 (2019). 30852123 10.1016/j.tics.2019.01.012 PMC6438863

[32] Bentz D. & Schiller D. Threat processing: models and mechanisms. WIREs Cognitive Science 6, 427–439 (2015). 26267313 10.1002/wcs.1353

[33] Lee J. & Sabatini B. L. Striatal indirect pathway mediates exploration via collicular competition. Nature 599, 645–649 (2021). 34732888 10.1038/s41586-021-04055-4 PMC10281058

[34] Gray J. A. & McNaughton N. Comparison between the behavioural effects of septal and hippocampal lesions: A review. Neuroscience & Biobehavioral Reviews 7, 119–188 (1983). 6348604 10.1016/0149-7634(83)90014-3

[35] Sibole W., Miller J. J. & Mogenson G. J. Effects of septal stimulation on drinking elicited by electrical stimulation of the lateral hypothalamus. Experimental Neurology 32, 466–477 (1971). 5110228 10.1016/0014-4886(71)90012-4

[36] Azevedo E. P. A limbic circuit selectively links active escape to food suppression. eLife 9, e58894 (2020). 32894221 10.7554/eLife.58894 PMC7476759

[37] Shin S. Drd3 Signaling in the Lateral Septum Mediates Early Life Stress-Induced Social Dysfunction. Neuron 97, 195–208.e6 (2018). 29276054 10.1016/j.neuron.2017.11.040 PMC5766830

[38] Rodriguez L. A. TrkB-dependent regulation of molecular signaling across septal cell types. 2023.06.29.547069 Preprint at 10.1101/2023.06.29.547069 (2023). PMC10805920 38263132

[39] SimonR. C. Opioid-driven disruption of the septal complex reveals a role for neurotensin-expressing neurons in withdrawal. 2024.01.15.575766 Preprint at 10.1101/2024.01.15.575766 (2024).40378834

[40] Reid C. M. Multimodal classification of neurons in the lateral septum. 2024.02.15.580381 Preprint at 10.1101/2024.02.15.580381 (2024).

[41] Hughes A. C. A single-vector intersectional AAV strategy for interrogating cellular diversity and brain function. Nat Neurosci 27, 1400–1410 (2024). 38802592 10.1038/s41593-024-01659-7 PMC12955835

[42] Kepecs A. & Fishell G. Interneuron cell types are fit to function. Nature 505, 318–326 (2014). 24429630 10.1038/nature12983 PMC4349583

[43] Luo A. H., Tahsili-Fahadan P., Wise R. A., Lupica C. R. & Aston-Jones G. Linking Context with Reward: A Functional Circuit from Hippocampal CA3 to Ventral Tegmental Area. Science 333, 353–357 (2011). 21764750 10.1126/science.1204622 PMC3150711

[44] Goode T. 141. Calibration of Context-Evoked Feeding by a Genetically Defined Lateral Septum to Lateral Hypothalamus Circuit. Biological Psychiatry 93, S151 (2023).

[45] Tingley D. & Buzsáki G. Transformation of a Spatial Map across the Hippocampal-Lateral Septal Circuit. Neuron 98, 1229–1242.e5 (2018). 29779942 10.1016/j.neuron.2018.04.028 PMC6605060

[46] Tingley D. & Buzsáki G. Routing of Hippocampal Ripples to Subcortical Structures via the Lateral Septum. Neuron 105, 138–149.e5 (2020). 31784288 10.1016/j.neuron.2019.10.012 PMC6952543

[47] Mogg K. & Bradley B. P. Anxiety and Threat-Related Attention: Cognitive-Motivational Framework and Treatment. Trends in Cognitive Sciences 22, 225–240 (2018). 29402737 10.1016/j.tics.2018.01.001

[48] Sussman T. J., Jin J. & Mohanty A. Top-down and bottom-up factors in threat-related perception and attention in anxiety. Biological Psychology 121, 160–172 (2016). 27546616 10.1016/j.biopsycho.2016.08.006

[49] Bishop S. J. Neural Mechanisms Underlying Selective Attention to Threat. Annals of the New York Academy of Sciences 1129, 141–152 (2008). 18591476 10.1196/annals.1417.016

[50] Rauss K. & Pourtois G. What is Bottom-Up and What is Top-Down in Predictive Coding? Front Psychol 4, 276 (2013). 23730295 10.3389/fpsyg.2013.00276 PMC3656342

[51] Reardon T. R. Rabies Virus CVS-N2cΔG Strain Enhances Retrograde Synaptic Transfer and Neuronal Viability. Neuron 89, 711–724 (2016). 26804990 10.1016/j.neuron.2016.01.004 PMC4760870

[52] Sumser A., Joesch M., Jonas P. & Ben-Simon Y. Fast, high-throughput production of improved rabies viral vectors for specific, efficient and versatile transsynaptic retrograde labeling. eLife 11, e79848 (2022). 36040301 10.7554/eLife.79848 PMC9477495

[53] Zhang X. Genetically identified amygdala–striatal circuits for valence-specific behaviors. Nat Neurosci 24, 1586–1600 (2021). 34663958 10.1038/s41593-021-00927-0 PMC8556347

[54] Ma J. Divergent projections of the paraventricular nucleus of the thalamus mediate the selection of passive and active defensive behaviors. Nat Neurosci 24, 1429–1440 (2021). 34413514 10.1038/s41593-021-00912-7 PMC8484052

[55] Xiao X. A Genetically Defined Compartmentalized Striatal Direct Pathway for Negative Reinforcement. Cell 183, 211–227.e20 (2020). 32937106 10.1016/j.cell.2020.08.032 PMC8605319

[56] LeDuke D. O., Borio M., Miranda R. & Tye K. M. Anxiety and depression: A top-down, bottom-up model of circuit function. Annals of the New York Academy of Sciences n/a,. 10.1111/nyas.14997 PMC10695657 37129246

[57] Wong L. C. Effective Modulation of Male Aggression through Lateral Septum to Medial Hypothalamus Projection. Current Biology 26, 593–604 (2016). 26877081 10.1016/j.cub.2015.12.065 PMC4783202

[58] Leroy F. A circuit from hippocampal CA2 to lateral septum disinhibits social aggression. Nature 564, 213–218 (2018). 30518859 10.1038/s41586-018-0772-0 PMC6364572

[59] Prescott S. L., Umans B. D., Williams E. K., Brust R. D. & Liberles S. D. An Airway Protection Program Revealed by Sweeping Genetic Control of Vagal Afferents. Cell 181, 574–589.e14 (2020). 32259485 10.1016/j.cell.2020.03.004 PMC7197391

[60] Matho K. S. Genetic dissection of the glutamatergic neuron system in cerebral cortex. Nature 598, 182–187 (2021). 34616069 10.1038/s41586-021-03955-9 PMC8494647

[61] Rossi J. Melanocortin-4 receptors expressed by cholinergic neurons regulate energy balance and glucose homeostasis. Cell Metab 13, 195–204 (2011). 21284986 10.1016/j.cmet.2011.01.010 PMC3033043

[62] Williams E. K. Sensory Neurons that Detect Stretch and Nutrients in the Digestive System. Cell 166, 209–221 (2016). 27238020 10.1016/j.cell.2016.05.011 PMC4930427

[63] Pan W. Essential Role for Hypothalamic Calcitonin Receptor‒Expressing Neurons in the Control of Food Intake by Leptin. Endocrinology 159, 1860–1872 (2018). 29522093 10.1210/en.2017-03259 PMC5888224

[64] Rousso D. L. Two Pairs of ON and OFF Retinal Ganglion Cells Are Defined by Intersectional Patterns of Transcription Factor Expression. Cell Rep 15, 1930–1944 (2016). 27210758 10.1016/j.celrep.2016.04.069 PMC4889540

[65] Trusel M. Punishment-Predictive Cues Guide Avoidance through Potentiation of Hypothalamus-to-Habenula Synapses. Neuron 102, 120–127.e4 (2019). 30765165 10.1016/j.neuron.2019.01.025

[66] Weinreb C. Keypoint-MoSeq: parsing behavior by linking point tracking to pose dynamics. Nat Methods 21, 1329–1339 (2024). 38997595 10.1038/s41592-024-02318-2 PMC11245396

[67] Arch V. S. & Narins P. M. ‘Silent’ signals: selective forces acting on ultrasonic communication systems in terrestrial vertebrates. Animal Behaviour 76, 1423–1428 (2008). 19802327 10.1016/j.anbehav.2008.05.012 PMC2598741

[68] Kennedy A. Stimulus-specific hypothalamic encoding of a persistent defensive state. Nature 586, 730–734 (2020). 32939094 10.1038/s41586-020-2728-4 PMC7606611

[69] Hersman S., Allen D., Hashimoto M., Brito S. I. & Anthony T. E. Stimulus salience determines defensive behaviors elicited by aversively conditioned serial compound auditory stimuli. eLife 9, e53803 (2020). 32216876 10.7554/eLife.53803 PMC7190350

[70] Dinç F. Fast, scalable, and statistically robust cell extraction from large-scale neural calcium imaging datasets. 2021.03.24.436279 Preprint at 10.1101/2021.03.24.436279 (2024).

[71] Pachitariu M. Suite2p: beyond 10,000 neurons with standard two-photon microscopy. 061507 Preprint at 10.1101/061507 (2017).

[72] Burgess C. R. Hunger-Dependent Enhancement of Food Cue Responses in Mouse Postrhinal Cortex and Lateral Amygdala. Neuron 91, 1154–1169 (2016). 27523426 10.1016/j.neuron.2016.07.032 PMC5017916

